# Targeted Next-Generation Sequencing-Based Multiple Gene Mutation Profiling of Patients with Rectal Adenocarcinoma Receiving or Not Receiving Neoadjuvant Chemoradiotherapy

**DOI:** 10.3390/ijms231810353

**Published:** 2022-09-08

**Authors:** You-Kang Chang, Hui-Hwa Tseng, Chung-Man Leung, Kuo-Cheng Lu, Kuo-Wang Tsai

**Affiliations:** 1 Department of Radiation Oncology, Taipei Tzu Chi Hospital, Buddhist Tzu Chi Medical Foundation, Taipei 23142, Taiwan; 2 College of Medicine, Tzu Chi University, Hualien City 97004, Taiwan; 3 Department of Anatomic Pathology, Taipei Tzu Chi Hospital, Buddhist Tzu Chi Medical Foundation, New Taipei City 97004, Taiwan; 4 Department of Radiation Oncology, Kaohsiung Veterans General Hospital, Kaohsiung 81341, Taiwan; 5 Division of Nephrology, Department of Medicine, Taipei Tzu Chi Hospital, Buddhist Tzu Chi Medical Foundation, New Taipei City 97004, Taiwan; 6 Division of Nephrology, Department of Medicine, Fu-Jen Catholic University Hospital, School of Medicine, Fu-Jen Catholic University, New Taipei City 24205, Taiwan; 7 Department of Research, Taipei Tzu Chi Hospital, Buddhist Tzu Chi Medical Foundation, New Taipei City 23142, Taiwan

**Keywords:** rectal carcinoma, cancer panel, next-generation sequencing, chemoradiotherapy

## Abstract

This study investigated whether oncogenic and tumor-suppressive gene mutations are involved in the differential outcomes of patients with rectal carcinoma receiving neoadjuvant chemoradiotherapy (nCRT). Genomic DNA was obtained from formalin-fixed paraffin-embedded (FFPE) specimens of patients with rectal carcinoma who received a complete nCRT course. Gene mutation status was examined in specimens from patients before and after nCRT by using the AmpliSeq platform. Our data revealed that the nonsynonymous *p53*, *APC*, *KRAS*, *CDKN2A*, and *EGFR* mutations were observed in 93.1%, 65.5%, 48.6%, and 31% of the patients with rectal adenocarcinoma, respectively. *BRAF*, *FBXW7*, *PTEN*, and *SMAD4* mutations were observed in 20.7% of patients with rectal carcinoma. The following 12 gene mutations were observed more frequently in the patients exhibiting a complete response than in those demonstrating a poor response before nCRT: *ATM*, *BRAF*, *CDKN2A*, *EGFR*, *FLT3*, *GNA11*, *KDR*, *KIT*, *PIK3CA*, *PTEN*, *PTPN11*, *SMAD4*, and *TP53*. In addition, *APC*, *BRAF*, *FBXW7*, *KRAS*, *SMAD4*, and *TP53* mutations were retained after nCRT. Our results indicate a complex mutational profile in rectal carcinoma, suggesting the involvement of *BRAF*, *SMAD4*, and *TP53* genetic variants in the outcomes of patients with nCRT.

## 1. Introduction

Rectal carcinoma is a common cause of cancer deaths worldwide. More than 0.73 million new rectal cancer cases and 339,022 deaths were estimated to occur in 2020, representing about 3.4% of cancer cases and deaths [1]. Preoperative neoadjuvant chemoradiotherapy (nCRT) is a general therapeutic modality for patients with rectal adenocarcinoma and locally advanced rectal cancer, and has been demonstrated to significantly reduce local recurrence and prolong survival [2,3]. However, many patients who receive nCRT experience no benefit but severe side effects [4,5]. Until now, no precision biomarkers for predicting patients who would have a complete response following nCRT have been identified. DNA alterations, abnormal gene expression, and epigenetic changes can be used as biomarkers for predicting the tumor response to radiotherapy in patients with rectal carcinoma [6,7,8]. Genetic mutations were identified as the predictors of the tumor response to nCRT in rectal cancer, including *TP53*, *KRAS*, and *EGFR* [9,10,11]. Although many genetic mutations have been identified as the biomarkers of the rectal tumor response to nCRT in rectal cancer, some studies have reported opposite results or indicated that genetic mutations cannot be used as predictors [12,13,14].

The Sanger sequencing method is a gold standard for identifying gene variants in cancer; however, it has low throughput and poor sensitivity [15]. A powerful method, next-generation sequencing (NGS) with high throughput, was developed to comprehensively identify gene mutations in the whole genome in patients with cancer [16,17,18]. Whole-genome sequencing is expensive because only 3–4% of the genome comprises the protein-coding region [19]. The exon capture method for targeted sequencing, or targeting a subset of genes of interest for sequencing, has been widely used and can reduce costs and time. Until now, targeted amplicon-based multigene mutational screening has been widely applied for the detection of gene variants in patients with cancer; this method can use a minimum amount of gDNA from a formalin-fixed paraffin-embedded (FFPE) sample [16,20,21,22]. In this study, we examined 55 samples from 29 patients with rectal carcinoma. All the 29 patients received preoperative chemoradiotherapy, and 26 of the 29 patients received postoperative chemoradiotherapy. Identification of gene mutations in patients receiving preoperative nCRT may help determine mutations driving rectal carcinoma progression. Moreover, identification of gene mutations in patients receiving postoperative nCRT may help identify those involved in resistance to nCRT. Our findings provide new insights to evaluate the response of advanced rectal cancer to nCRT.

## 2. Results

**Figure 1:**
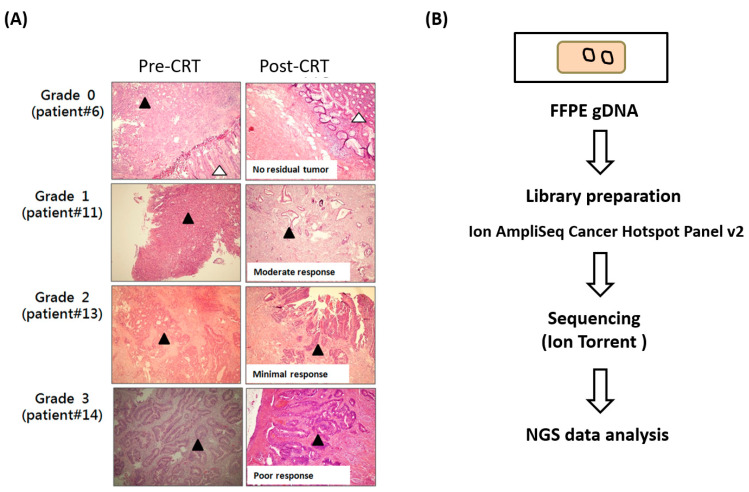
Identification of gene variants in rectal carcinoma through NGS. (**A**) Histopathological examination of biopsy specimens collected from patients with rectal carcinoma before nCRT (left panels). Histopathological examination of surgery specimens collected from patients with rectal carcinoma after nCRT (right panels). The black triangle indicates tumor cells, and the white triangle indicates the normal tissue. The response to nCRT was determined on the basis of the tumor regression grade. The image of specimens was determined through the microscope (magnification with 40× lens). (**B**) Targeted NGS workflow. Ten nanograms of genomic DNA was used for AmpliSeq cancer panel library preparation. Ion and high-throughput sequencing was performed using ion Torrent.

This study included 29 patients with rectal carcinoma who received a complete nCRT course before surgery. Before nCRT, 29 FFPE rectal carcinoma specimens were collected from the biopsy samples of the patients. Following complete nCRT, we collected the corresponding post-nCRT FFPE surgical specimens from these patients. Table 1 summarizes the clinicopathological features of the patients. Among the 29 patients, 9, 11, and 9 exhibited a complete, partial, and poor response to nCRT (tumor regression grades 0, 1, and 2 or 3), respectively (Figure 1). To identify whether gene variant profiles differ between the patients exhibiting a complete response and those exhibiting a poor response to nCRT, we performed the NGS of 50 genes in the 55 specimens obtained from the 29 patients with rectal carcinoma. We extracted genomic DNA from the FFEP specimens and amplified them using 207 primer pairs to amplify the hotspot regions of 50 genes (Figure 1B). Amplicons were identified through NGS, and gene variants were analyzed by performing a bioinformatics analysis. Three types of surgical specimens were collected from 21, 26, and 28 patients after nCTR failed to meet quality control requirements (Supplementary Table S1). Furthermore, an average of 1,379,426 mapped sequence reads were obtained for the 55 samples, and the 500× coverage was 98.6% (Supplementary Table S1).

**Figure 2:**
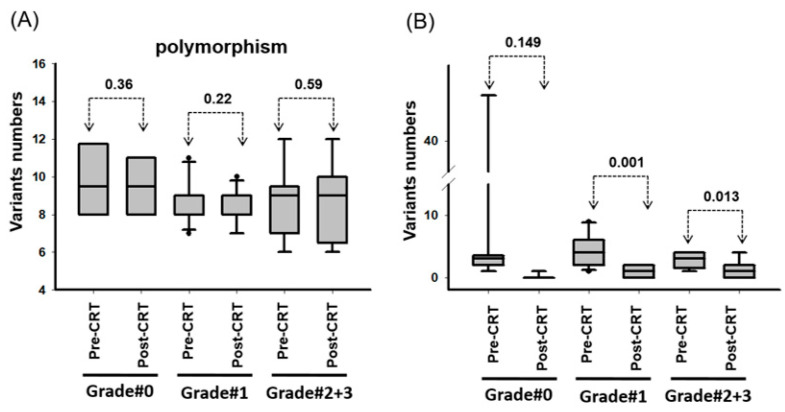
Genetic variants were analyzed in biopsy specimens from 29 patients with rectal carcinoma before and after nCRT. (**A**) The number of genes with polymorphisms were detected in an individual patient before and after nCRT. (**B**) The numbers of gene mutations were detected in an individual patient before and after nCRT. The variants with an allelic frequency of >5% and a variant count of >25 were included.

Through sequencing, we identified 648 variants (frequency > 5%) in 50 genes, including 297 nonsynonymous and 351 synonymous variants (Supplementary Tables S2–S4). Furthermore, we determined that the number of single nucleotide polymorphism (SNP) did not significantly differ between the pre-nCRT and post-nCRT specimens (Figure 2A). The number of gene mutations was lower in the post-CRT specimens than in the pre-CRT specimens (Figure 2B).

To identify gene mutations involved in nCRT resistance, we excluded SNPs by using the SNP database. As presented in Supplementary Table S5, a total of 160 mutations were identified with a frequency of >5% in the 55 rectal carcinoma specimens, including 30 synonymous and 130 nonsynonymous variants. Because nonsynonymous variants considerably affect gene function, we included rare mutations with frequencies ranging from 2% to 5%. As presented in Supplementary Table S4, we identified 394 mutations with a frequency of 2–5% from all the samples. These gene candidates with nonsynonymous mutations (>2%) were included in the subsequent analysis.

**Figure 3:**
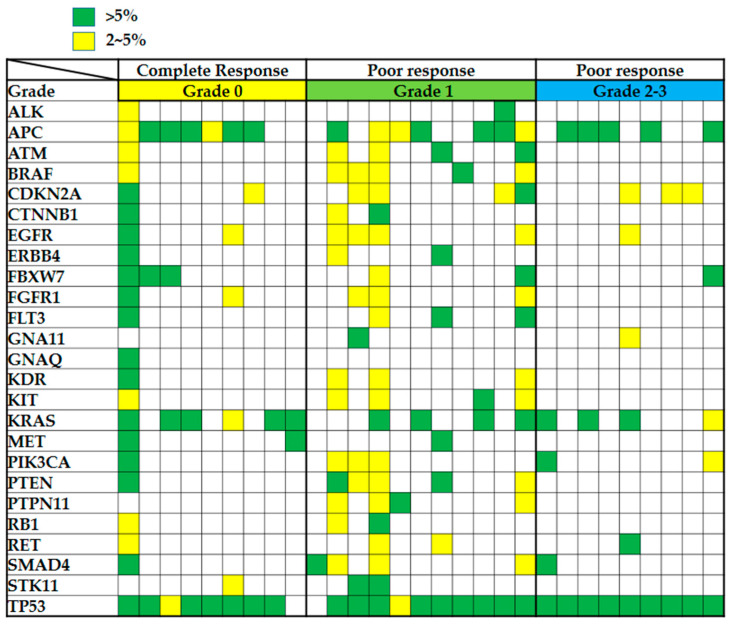
Gene mutation status was examined in biopsy samples from patients with rectal carcinoma. Mutation profiles of biopsy specimens collected from 29 patients with rectal carcinoma before CRT, including 9 patients with grade 0, 11 patients with grade 1, and 9 patients with grade 2 or 3. A green square indicates that a mutation with a frequency of >5% was detected in the gene, a yellow square indicates that a mutation with a frequency of 2–5% was observed in the gene, and an empty square indicates that no relevant mutation was observed for the gene.

As presented in Figure 3 and Table 2, the top 10 high-frequency nonsynonymous genes mutations, *TP53* (27/29; 93.1%), *APC* (19/29; 65.5%), *KRAS* (14/29; 48.3%), *CDKN2A* (9/29; 31%), *EGFR* (7/29; 24.1%), *FBXW7* (6/29; 20.7%), *BRAF* (6/29; 20.7%), *SMAD4* (6/29; 20.7%), *PIK3CA* (6/29; 20.7%), and *PTEN* (6/29; 20.7%), were identified in the pre-nCRT biopsy specimens. These variants resulted in nonsynonymous changes in protein sequences. We compared the mutation frequency between the patients exhibiting a complete response (tumor regression grade 0) and those demonstrating a poor response (tumor regression grades 1–3). We identified a higher mutation frequency of the following 12 genes in the complete response group than in the poor response group after nCRT treatment: *ATM* (grade 0: 11.1%; grade 1–3: 20%), *BRAF* (grade 0: 11.1%; grade 1–3: 25%), *CDKN2A* (grade 0: 22.2%; grade 1–3: 35%), *EGFR* (grade 0: 22.2%; grade 1–3: 25%), *FLT3* (grade 0: 11.1%; grade 1–3: 15%), *GNA11* (grade 0: 0%; grade 1–3: 10%), *KDR* (grade 0: 11.1%; grade 1–3: 15%), *KIT* (grade 0: 11.1%; grade 1–3: 20%), *PIK3CA* (grade 0: 11.1%; grade 1–3: 25%), *PTEN* (grade 0: 11.1%; grade 1–3: 25%), *PTPN11* (grade 0: 11.1%; grade 1–3: 20%), *SMAD4* (grade 0: 11.1%; grade 1–3: 25%), and *TP53* (grade 0: 88.9%; grade 1–3: 95%); Figure 3 and Table 2. Due to the small sample size, these gene mutations were not significantly different in poor CRT response compared to complete response by chi-square test (Supplementary Table S6).

**Figure 4:**
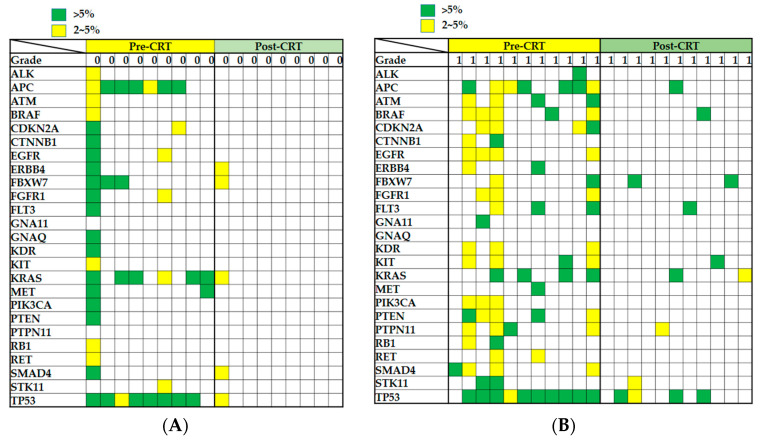

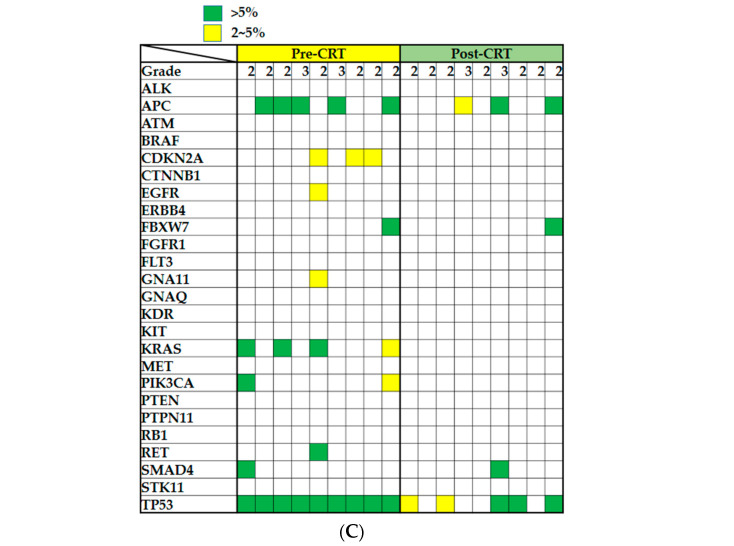
Gene mutation status was examined in specimens from patients with rectal carcinoma before and after nCRT. (**A**) Mutation profiles of biopsy-collected specimens (pre-nCRT) and surgery-collected specimens (post-nCRT) from nine patients with rectal carcinoma with tumor regression grade 0. (**B**) Mutation profiles of biopsy-collected specimens (pre-nCRT) and surgery-collected specimens (post-nCRT) from 11 patients with rectal carcinoma with tumor regression grade 1. (**C**) Mutation profiles of biopsy-collected specimens (pre-nCRT) and surgery-collected specimens (post-nCRT) from nine patients with rectal carcinoma with tumor regression grade 2–3. A green square indicates that a mutation with a frequency of >5% was detected in a gene, a yellow square indicates that a mutation with a frequency of 2–5% was observed in a gene, and an empty square indicates that no relevant mutation was observed for the gene.

After the excision of the tumor, gene mutations should be gradually decreased during the nCRT process, especially in patients exhibiting a complete response. If a gene mutation contributes to nCRT resistance in rectal carcinoma, it should be retained after nCRT. Our data revealed that most of the mutations disappeared after the completion of nCRT in the patients exhibiting a complete response (Figure 4A). We compared the mutation status between the pre-nCRT and post-nCRT specimens in the patients exhibiting a poor response and observed that some of the gene mutations were retained, namely *APC*, *BRAF*, *FBXW7*, *FLT3*, *KIT*, *KRAS*, *PTPN11*, *SMAD4*, *STK11*, and *TP53* (Figure 4B,C and Table 3). Among them, three gene mutations, *BRAF*, *SMAD4*, and *TP53*, were more frequently observed in the poor response group than in the complete response group (Figure 3 and Table 2). On the basis of these complex mutational profiles in rectal carcinoma, we speculated about the involvement of *BRAF*, *SMAD4*, and *TP53* gene mutations in the outcomes of nCRT in patients with rectal carcinoma.

**Figure 5:**
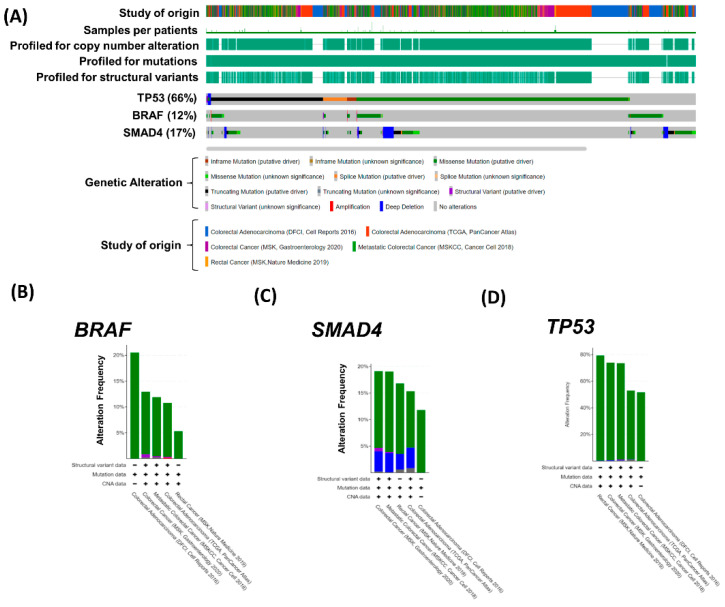

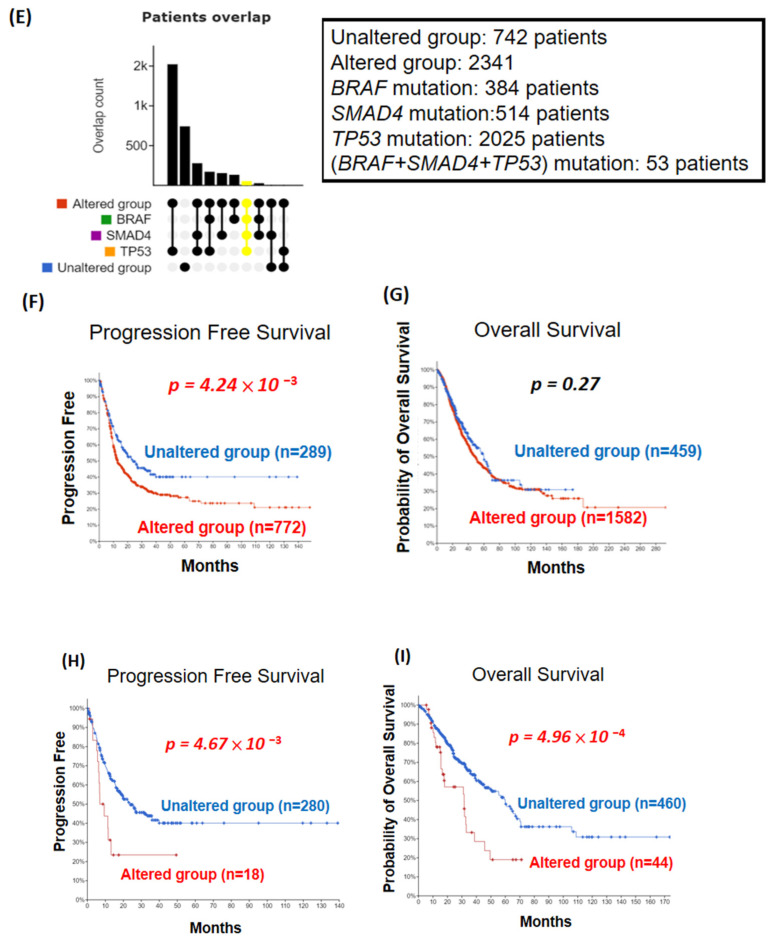
*BRAF*, *SMAD4*, and *TP53* genetic variants in patients with colorectal carcinoma. (**A**) Oncoprint indicates genetic alterations *in BRAF*, *SMAD4,* and *TP53* in patients with colorectal carcinoma, respectively. Colors indicate the type of genetic alterations (green: mutation; purple: structure variation; red: amplification, and gray: multiple alterations) and different cohorts below the oncoprint. (**B**–**D**) Alteration frequency of *BRAF*, *SMAD4*, and *TP53* in colorectal carcinoma in five cohorts. CAN = copy number alterations. (**E**) Distribution of patients with *BRAF*, *SMAD4*, and *TP53* genetic variants. (**F**,**G**) The effect of variants in *BRAF*, *SMAD4*, or TP53 on progression-free survival and overall survival were analyzed from five colorectal cancer databases. (**H**,**I**) The effects of the simultaneous occurrence of *BRAF*, *TP53*, and *SMAD4* mutations on progression-free survival and overall survival were analyzed from five rectal cancer databases.

We investigated the effects of *BRAF*, *SMAD4*, and *TP53* mutations on colorectal carcinoma progression by using cBioPortal for Cancer Genomics (http://cbioportal.org, accessed on 3 March 2021) [23,24]. The mutation profiles of 3083 patients with colorectal carcinoma were downloaded from five studies [25,26,27,28]. The mutation rates of *BRAF*, *SMAD4*, and *TP53* were 12.4% (384 patients), 16.6% (514 patients), and 65.6% (2025 patients) in the colorectal carcinoma cohort, respectively (Figure 5A–E). Furthermore, mutations in *BRAF*, *TP53*, or *SMAD4* significantly reduced the progression-free survival of patients with colorectal carcinoma (*p* = 4.24 × 10^−4^) but were not correlated with overall survival (*p* = 0.27; Figure 5F,G). The simultaneous occurrence of *BRAF*, *TP53*, and *SMAD4* mutations significantly reduced the progression-free survival (*p* = 4.67 × 10^−3^) and overall survival (*p* = 4.98 × 10^−4^) of patients with colorectal cancer (Figure 5H,I). Mutations in *BRAF*, *SMAD4*, or *TP53* were significantly correlated with the poor progression-free survival (*BRAF*: *p* = 3.62 × 10^−3^; *SMAD4*: *p* = 6.28 × 10^−4^; and *TP53*: *p* = 6.33 × 10^−5^) and overall survival (*BRAF*: *p* = 9.96 × 10^−9^; *SMAD4*: *p* = 5.96 × 10^−3^) of patients with colorectal cancer. However, the *TP53* mutation alone was not correlated with poor overall survival (Supplementary Figure S1). In general, patients with advanced rectal cancer will be treated with nCRT before surgery. Therefore, a cohort of metastatic colorectal cancer has further analyzed the prognosis of BRAF, SMAD4, and TP53 variants. Similar results revealed that mutations in *BRAF* and *SMAD4* were significantly correlated with the overall survival (Supplementary Figure S2; *BRAF*: *p* = 4.47 × 10^−6^ and *SMAD4*: *p* = 0.048) of patients with metastatic colorectal cancer. However, the *TP53* mutation alone was not correlated with poor overall survival of patients with metastatic colorectal carcinoma.

Taken together, our results indicate that nCRT might result in physiological selective pressure to accumulate gene mutations in residual rectal cancer regions. *BRAF*, *SMAD4*, and *TP53* genetic mutations might be involved in resistance to nCRT and poor prognosis in colorectal carcinoma. However, these findings must be confirmed. 

## 3. Discussion

Preoperative nCRT followed by tumor resection has become the standard treatment guideline for patients with locally advanced rectal cancer. Preoperative nCRT can downstage the tumor and increase the possibility of sphincter preservation [29]. Such treatments can enable patients to have a better quality of life after surgery. Although numerous studies have reported that several genetic mutations can serve as the biomarkers of the response of the rectal tumor to nCRT, some studies have indicated opposite results or that genetic mutations cannot be used as predictors. Yang et al. performed the whole-genome sequencing of 28 paired advanced rectal cancer specimens before and after CRT and observed that mutations in *CTDSP2*, *APC*, *KRAS*, *TP53*, and *NFKBIZ* confer selective pressure on cancer cells, resulting in resistance to CRT [12]. Concurrent *KRAS* and *TP53* mutations contributed to resistance to CRT and metastasis in rectal cancer [13]. A study examining the genetic profiles of 229 pretreatment specimens from patients with stage II or III rectal cancer reported that *KRAS* and combined *KRAS*/*TP53* mutations acted as independent biomarkers for a poor response to nCRT [14]. Garcia-Aguilar et al. reported that three individual genetic mutations, *KRAS*, *CCND1*, and *MTHFR*, were well correlated with a complete response (grade 0) to CRT in rectal carcinoma [30]. However, *BRAF* mutations were not detected, and *KRAS* and *PTEN* mutations were reported to not be associated with a response to cetuximab-based chemoradiotherapy [31]. Davies et al. reported that *ERK* and *AKT* signaling activation but not *KRAS* mutations were well correlated with the response to nCRT in rectal carcinoma [32]. Compared with wild-type *KRAS*, *KRAS* mutations were associated with a poor response to nCRT, especially the *KRAS* codon 13 mutation. In addition, tumors with the *KRAS* codon 13 mutation are often accompanied by *TP53* mutations [8]. Russo et al. observed a high frequency of mutations in *KRAS* (43%), *APC* (17%), *BRAF* (4%), *NRAS* (4%), *PIK3CA* (4%), and *TP53* (11%) in rectal cancer, and these mutations were well associated with a complete response to nCRT, especially in patients with *BRAF*, *NRAS*, *APC*, or *TP53* mutations [33]. These findings indicate that *TP53*, *KRAS*, and *BRAF* mutations might be used as biomarkers for the prediction of the response to nCRT. 

Russo et al. did not observe differences in individual gene mutation rates between pre- and post-CRT samples [33]. We used the pre-nCRT and post-nCRT paired samples to identify gene mutation biomarkers for predicting the response to nCRT. Our findings are consistent with those reported by Russo. We observed that most of the gene mutations disappeared after nCRT (Figure 4). Some genetic mutations were retained after nCRT, suggesting that these gene mutations might contribute to resistance to nCRT. Finally, three genetic mutations, *BRAF*, *SMAD4*, and *TP53*, were successfully identified as biomarkers for predicting the response to nCRT. By examining the mutation status of *BRAF*, *SMAD4*, and *TP53*, we can identify patients who would benefit from nCRT and select alternative therapeutic strategies for those who would not benefit from nCRT. In addition, we noted that *BRAF* genetic mutations exhibited mutual exclusivity with *TP53* mutations in rectal cancer but significantly cooccurred (*p* < 0.001) with *SMAD4* genetic mutations (*p* = 0.024).

We identified that mutations in *BRAF*, *SMAD4*, and *TP53* might contribute to the response to nCRT in patients with rectal carcinoma. Among them, *SMAD4* mutations have been rarely identified in other studies through high-throughput sequencing to identify biomarkers of the response to nCRT. A study reported that *SMAD4* mutations or deletions frequently occurred in late-stage colon cancer [34]. Another study reported that the loss of expression of *SMAD4*-induced *RICTOR/AKT* signaling activation was correlated with the poor survival of patients with colorectal carcinoma [35]. Furthermore, the depletion of *SMAD4* expression or the activation of *RICTOR/AKT* signaling contributed to resistance to irinotecan in colon cancer cells. In addition, some studies have reported that the loss of *SMAD4* expression contributed to resistance to 5-fluorouracil in colon cancer [36,37]. *SMAD4* expression causes radioresistance in pancreatic cancer through the induction of reactive oxygen species and autophagy [38]. A meta-analysis revealed that *SMAD4* mutations were well associated with overall, progression-free, and relapse-free survival and several clinicopathological parameters, including lymph node metastasis [39]. These findings suggest that *SMAD4* mutations are involved in sensitivity to a chemotherapeutic drug and cancer progression. *TP53* is a crucial tumor suppressor involved in maintaining genome stability and regulating the cell cycle and apoptosis. Park et al. reported that the loss of *SMAD4* and *TP53* synergistically occurs in intestinal carcinogenesis by inhibiting *p21* and increasing *Wnt/β-catenin* signaling activity [40]. Although oncogenic *BRAF* mutations play a crucial role in tumorigenesis, only the *BRAF* mutant was inefficient in generating tumors in vivo. Loss of pro-differentiation transcription factors, such as *CDX2* and *SMAD4*, can accelerate tumorigenesis [41,42]. Taken together, these findings indicate that *SMAD4* is a crucial tumor suppressor and that its mutation might be involved in several cancer cell biological functions, including maintaining cancer genome stability and regulating the cell cycle and apoptosis. The results demonstrate that *SMAD4* mutations might serve as a biomarker for predicting the response of rectal cancer to nCRT. However, more data need to be collected prospectively to further demonstrate these new findings.

Although our study identified three genetic variant genes (*BRAF*, *SMAD4*, and *TP53*) that may act as biomarkers for predicting nCRT response, there were some shortcomings in our study. First, the small sample size resulted in low statistical power. In addition, the study relied on retrospective data and specimens. It might have missed some important information, and the nCRT process might be slightly different for each patient. These shortcomings resulted in most gene variants showing no significant difference between patients with good and poor responses to nCRT (Supplementary Table S6). In order to overcome the small sample size, we attempted to evaluate the clinical impacts of *BRAF*, *SMAD4*, and *TP53* mutation by analyzing publicly available datasets. Combined with public datasets, it was further confirmed that patients with *BRAF*, *SMAD4,* and *TP53* gene variants were significantly associated with poorer progression-free survival in colorectal cancer. Although combining data from various databases can improve statistical power, differences in genetic backgrounds by race and lack of standard treatment procedures for each patient remain issues to overcome.

In summary, we observed that the patients whose tumors harbored *BRAF*, *SMAD4*, and *TP53* genetic mutations had significantly poorer disease-free survival, and the detection of these mutations can help identify patients who would benefit from nCRT. Detection of these genetic biomarkers can enable the selection of optimal treatment strategies and improvement in patients’ quality of life after surgery.

## 4. Methods and Materials

### 4.1. Clinical Samples

Patients were diagnosed with histologically confirmed rectal cancer between 2014 and 2018. Before any treatment, all cases underwent endorectal ultrasonography and biopsy. All patients needed to conduct a complete nCRT therapy course before surgery. FFPE rectal cancer specimens from 29 patients were collected in this study, including pre-nCRT and corresponding post-nCRT FFPE specimens. These pre-nCRT and post-nCRT FFPE blocks were cut into 4-μm-thick sections for DNA extraction. Among them, the DNA extraction of post-nCRT FFPE specimens from 3 patients with grade 0 (complete response) failed due to too few tumor cells. Our study protocol was independently reviewed and approved by the Institutional Review Board of Kaohsiung Veterans General Hospital and Taipei Tzu Chi Hospital, Buddhist Tzu Chi Medical Foundation, (IRB approval number: VGHKS11-CT12-08 and 10-X-018).

### 4.2. DNA Extraction

Each FFPE block was cut into 4-μm-thick sections by using standard techniques. After excess wax was removed, pure tumor tissues were collected from three to four paraffin sections. Genomic DNA was extracted using the NucleoSpin FFPE DNA kit (Macherey-Nagel GmbH & Co. KG, Duren, Germany). The quantity and integrity of the extracted genomic DNA were determined using Qubit (Thermo Fisher Scientific Inc., San Jose, CA, USA) and Fragment Analyzer (Advanced Analytical Technologies, Inc., Ankeny, IA, USA), respectively.

### 4.3. Cancer Hotspot Panel v2 Sequencing

Ten nanograms of genomic DNA was amplified using 207 primer pairs (Ion AmpliSeq Cancer Hotspot Panel v2, Thermo Fisher Scientific Inc., San Jose, CA, USA) to target the hotspot regions of 50 genes. Amplicons were ligated with barcoded adaptors by using the Ion Amplicon Library Kit (Thermo Fisher Scientific Inc., San Jose, CA, USA). Barcoded libraries were subsequently conjugated with sequencing beads through emulsion polymerase chain reaction and enriched using IonChef (Thermo Fisher Scientific Inc., San Jose, CA, USA) in accordance with the Ion Torrent protocol (Thermo Fisher Scientific Inc., San Jose, CA, USA). The quality and quantity of the amplified library were determined using the fragment analyzer (AATI) and Qubit (Thermo Fisher Scientific Inc., San Jose, CA, USA), respectively. Sequencing was performed on the Ion Proton sequencer by using the Ion PI chip (Thermo Fisher Scientific Inc., San Jose, CA, USA) in accordance with the manufacturer’s protocol.

### 4.4. Data Analysis 

Raw reads generated by the sequencer were mapped to the hg19 reference genome by using the Ion Torrent Suite (v. 4.4). The coverage depth was calculated using the Torrent Coverage Analysis plug-in, and the result is presented in Supplementary Table S1. Single-nucleotide variants (SNVs) and short insertion/deletions were identified using the Torrent Variant Caller plug-in (version 4.4). The Variant Effect Predictor was used to annotate every variant with a database from COSMIC: v.70; dbSNP 138 and 1000 Genomes: phase 1. We filtered out variants with a coverage of lower than 50 or a frequency of <2%. Supplementary Tables S2–S4 list the results of gene variants detected through NGS.

### 4.5. Statistical Analysis

The variants numbers between pre-CRT and post-CRT were analyzed using Student’s t-tests. The demographics of patients with rectal cancer are presented as numbers and percentages. The chi-squared test was used to test the association between the categorical descriptive variables. Cumulative overall survival or progression free survival curves were estimated using the Kaplan–Meier method. The difference was considered significant when *p* < 0.05. All statistical analyses were performed using SPSS version 20.0 for Windows (SPSS Inc., Armonk, NY, USA).

## Figures and Tables

**Table 1:** Clinicopathological characteristics of 29 patients with rectal carcinoma.

	Tumor Regression						
	Grade 0 (*n* = 9)		Grade 1 (*n* = 11)		Grade 2–3 (*n* = 9)		
	*n*	(%)	*n*	(%)	*n*	(%)	*p* Value
Age							0.870
<65 years	4	(44.4)	6	(54.5)	5	(55.6)	
≥65 years	5	(55.6)	5	(45.5)	4	(44.4)	
Sex							0.568
Female	4	(44.4)	3	(27.3)	2	(22.2)	
Male	5	(55.6)	8	(72.7)	7	(77.8)	
Clinical stage							0.402
I-II	2	(22.2)	4	(36.4)	1	(11.1)	
III-IV	7	(77.8)	7	(63.6)	8	(88.9)	
pT stage							0.192
I-II	0	(0.0)	2	(18.2)	2	(22.2)	
III-IV	9	(100.0)	9	(81.8)	7	(77.8)	
pN stage							0.367
N0	3	(33.3)	4	(36.4)	1	(11.1)	
>N1	6	(66.7)	7	(63.6)	8	(88.9)	
pM stage							0.081
M0	7	(77.8)	11	(100.0)	9	(100.0)	
M1	2	(22.2)	0	(0.0)	0	(0.0)	
Lymph node metastasis							0.096
No	8	(88.9)	5	(45.5)	5	(55.6)	
Yes	1	(11.1)	6	(54.5)	4	(44.4)	

**Table 2:** Percentage of individual genes with mutations in patients with rectal carcinoma before and after nCRT.

	Total		Grade 0		Grade 1–3	
Gene	*n* = 29	%	*n* = 9	%	*n* = 20	%
ALK	2	6.9	1	11.1	1	5.0
APC	19	65.5	7	77.8	12	60.0
ATM	5	17.2	1	11.1	4	20.0
BRAF	6	20.7	1	11.1	5	25.0
CDKN2A	9	31.0	2	22.2	7	35.0
CTNNB1	3	10.3	1	11.1	2	10.0
EGFR	7	24.1	2	22.2	5	25.0
ERBB4	3	10.3	1	11.1	2	10.0
FBXW7	6	20.7	3	33.3	3	15.0
FGFR1	5	17.2	2	22.2	3	15.0
FLT3	4	13.8	1	11.1	3	15.0
GNA11	2	6.9	0	0.0	2	10.0
GNAQ	1	3.4	1	11.1	0	0.0
KDR	4	13.8	1	11.1	3	15.0
KIT	5	17.2	1	11.1	4	20.0
KRAS	14	48.3	6	66.7	8	40.0
MET	3	10.3	2	22.2	1	5.0
PIK3CA	6	20.7	1	11.1	5	25.0
PTEN	6	20.7	1	11.1	5	25.0
PTPN11	4	13.8	0	0.0	4	20.0
RB1	3	10.3	1	11.1	2	10.0
RET	4	13.8	1	11.1	3	15.0
SMAD4	6	20.7	1	11.1	5	25.0
STK11	3	10.3	1	11.1	2	10.0
TP53	27	93.1	8	88.9	19	95.0

**Table 3:** Gene mutations retained in rectal carcinoma after nCRT.

Patients No.	Genes	Protein Change	Mutation Type	Nucleotide Change	Pre-CRT%	Post-CRT%
23	APC	p.M1383fs	Frameshift	c.4146_4147insA	38.3	8.1
29	APC	p.L1488fs	Frameshift	c.4461delT	11.7	8.0
18	APC	p.E1309 *	Stop gained	c.3925G > T	16.6	6.6
18	APC	p.E1353 *	Stop gained	c.4057G > T	19.2	6.4
25	BRAF	p.V600E	Missense	c.1799T > A	15.1	11.9
7	FBXW7	p.G459E	Missense	c.1376G > A	0.0	5.2
29	FBXW7	p.R505C	Missense	c.1513C > T	12.2	9.6
23	KRAS	p.A146T	Missense	c.436G > A	21.7	5.1
18	SMAD4	p.A118V	Missense	c.353C > T	0.0	6.1
23	TP53	p.M237I	Missense	c.711G > A	37.6	6.9
25	TP53	p.R342 *	Stop gained	c.1024C > T	39.7	22.7
20	TP53	p.R213fs	Frameshift	c.636dupT	15.6	7.1
29	TP53	p.Q104 *	Stop gained	c.310C > T	23.6	12.0
18	TP53	p.V173M	Missense	c.517G > A	26.3	7.2

* indicates stop codon.

## Supplementary Materials

The following supporting information can be downloaded at: https://www.mdpi.com/article/10.3390/ijms231810353/s1.
