# Supplementary material for: Targeted Next-Generation Sequencing-Based Multiple Gene Mutation Profiling of Patients with Rectal Adenocarcinoma Receiving or Not Receiving Neoadjuvant Chemoradiotherapy
Source: Int J Mol Sci. 2022 Sep 8;23(18):10353. doi: 10.3390/ijms231810353 (PMC9499649; doi:10.3390/ijms231810353)
Supplement: Supplementary file 1 [file ijms-23-10353-s001.zip › ijms-1910530-supplementary.pdf]

Targeted Next-Generation Sequencing-Based Multiple Gene Mutation Profiling of Patients with Rectal Adenocarcinoma Receiving or Not Receiving Neoadjuvant Chemoradiotherapy

You-Kang Chang [1,2,†], Hui-Hwa Tseng [3,†], Chung-Man Leung [4], Kuo-Cheng Lu [5,6] and Kuo-Wang Tsai [7,*]

Supplementary Figure and Table

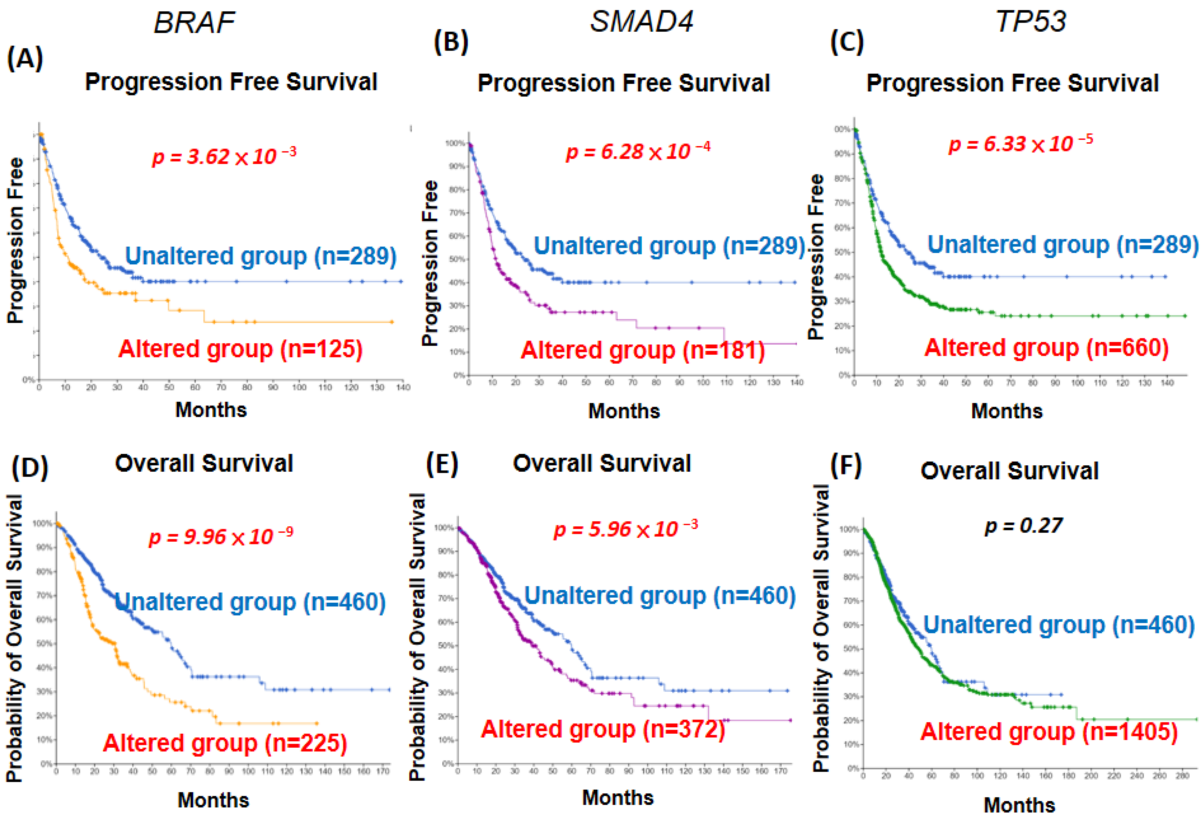

Supplementary Figure S1. *BRAF*, *SNAD4*, and *TP53* genetic variants as potential biomarkers for the prediction of colorectal carcinoma progression. (**A**–**C**) The effects of individual mutations in *BRAF*, *SMAD4*, and *TP53* on progression-free survival were analyzed from five rectal cancer databases. (**D**–**F**) The effects of *BRAF*, *TP53*, and *SMAD4* gene mutations on overall survival were analyzed from five rectal cancer databases.

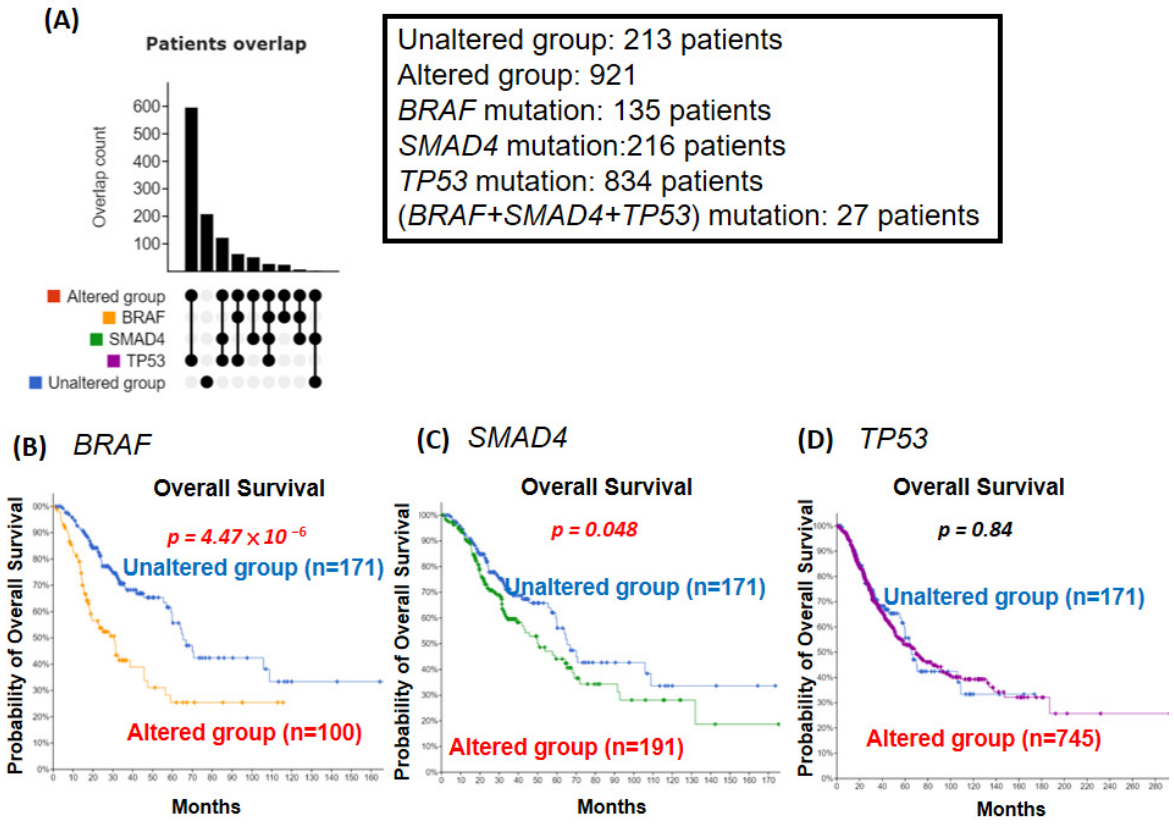

Supplementary Figure S2. *BRAF*, *SNAD4*, and *TP53* genetic variants as potential biomarkers for the prediction of metastatic colorectal carcinoma progression. (**A**) Distribution of patients with *BRAF*, *SMAD4*, and *TP53* genetic variants were shown from a metastatic colorectal cancer database. (**B**–**D**) The effects of *BRAF*, *SMAD4* and *TP53* gene mutations on OV were analyzed from a metastatic colorectal cancer database.

Supplementary Table S1. Information and sequencing of each sample

Tumor Regression grade	patients	Mapped Reads	Base On Target	Mean Depth	0.2X Coverage Uniformity	1x coverage	100x Coverage	500x Coverage
1	1A	1340456	0.943	5647	0.981	1	1	0.996
1	1B	1414280	0.963	6028	0.98	1	1	0.992
1	2A	1381298	0.96	6274	0.705	1	0.996	0.947
1	2B	1689017	0.979	7497	0.965	1	1	0.979
0	3A	652215	0.921	2593	0.892	1	0.987	0.908
0	3B	1119800	0.98	4770	0.969	1	1	0.991
0	4A	1522449	0.971	6649	0.958	1	1	0.985
0	4B	1249588	0.958	5199	0.963	1	1	0.982
2	5A	1596032	0.93	6676	0.969	1	1	0.996
2	5B	1448889	0.949	6105	0.958	1	1	0.992
0	6A	1540546	0.969	6664	0.988	1	1	1
0	6B	1608438	0.978	7075	0.978	1	1	0.996
1	7A	820219	0.983	3681	0.908	1	1	0.96
1	7B	988396	0.972	4170	0.941	1	1	0.992
0	8A	1190996	0.982	5120	0.888	1	0.99	0.951
0	8B	1285097	0.99	5728	0.956	1	1	0.994
1	9A	670708	0.791	2278	0.965	1	1	0.96
1	9B	1550568	0.967	6779	0.987	1	1	1
2	10A	1498237	0.983	6730	0.982	1	1	1

2	10B	1436602	0.977	6304	0.974	1	1	0.987
1	11A	1398115	0.983	6078	0.948	1	1	0.986
1	11B	1536484	0.966	6675	0.964	1	1	0.986
2	13A	1446717	0.965	6300	0.97	1	1	0.992
2	13B	1570842	0.988	6890	0.968	1	1	0.999
3	14A	1512508	0.979	6603	0.958	1	1	0.999
3	14B	1518553	0.985	6667	0.989	1	1	1
2	15A	1338787	0.959	5494	0.901	1	1	0.987
2	15B	1625771	0.969	7087	0.969	1	1	0.992
0	17A	1131082	0.979	4936	0.903	1	1	0.973
0	17B	1571168	0.958	6858	0.967	1	1	0.991
3	18A	1645076	0.981	7245	0.954	1	1	1
3	18B	1594615	0.967	7042	0.974	1	1	0.992
0	19A	630664	0.98	2675	0.945	1	1	0.965
0	19B	1287038	0.986	5676	0.942	1	1	0.99
2	20A	952894	0.945	3967	0.958	1	1	0.984
2	20B	1124015	0.974	4798	0.965	1	1	0.982
0	21A	889240	0.982	3797	0.941	1	1	0.965
0	21B*	24148	0.059	5.165	0.789	0.795	0	0
2	22A	1181665	0.958	4911	0.963	1	1	0.994
2	22B	1389732	0.981	6156	0.971	1	1	0.996
1	23A	1613549	0.981	7008	0.931	1	1	0.992

1	23B	947871	0.97	4036	0.956	1	1	0.981
1	24A	1330671	0.981	5809	0.947	1	1	0.994
1	24B	1650992	0.984	7325	0.967	1	1	0.996
1	25A	1252719	0.986	5467	0.905	1	1	0.971
1	25B	1588493	0.976	7079	0.978	1	1	0.996
0	26A	1772005	0.985	7849	0.914	1	1	0.997
0	26B*	1655	0.631	4.656	0.902	0.895	0	0
1	27A	1698283	0.99	7587	0.889	1	1	0.987
1	27B	1771937	0.95	7659	0.968	1	1	0.99
0	28A	1474727	0.981	6428	0.941	1	1	0.983
0	28B*	29302	0.094	12.03	0.448	0.662	0.013	0
2	29A	1646677	0.986	7236	0.945	1	1	0.996
2	29B	1618980	0.978	6941	0.967	1	1	0.999
1	30A	1925114	0.984	8442	0.907	1	1	0.998
1	30B	1936802	0.973	8426	0.979	1	1	0.996
1	31A	674067	0.963	2801	0.938	1	1	0.958
1	31B	1616735	0.974	6917	0.978	1	1	0.992

A: indicate specimens were collected by biopsy before nCRT

B: indicated specimens were collected by surgery after nCRT

***:gnomics DNA of these sample was poor quality resulted in failed to meet QC requirements**

Supplementary Table S2. Nonsynonymous variants of genes with more than 5% frequency identified in each library

Sample code	Chromosome coordinate					Variant information				Functional annotation	
	Gene symbol	Chr	Position	Ref	Var	Var freq	Var counts	Ref counts	Total depth	Protein change	consequence
1A	*FLT3*	13	2.9E+07	A	G	50.1%	1001	997	1998	-	splice_region_variant,intron_variant
1A	*KDR*	4	5.6E+07	T	A	100.0%	1976	0	1976	p.Q472H	missense_variant
1A	*TP53*	17	7579472	G	C	49.4%	978	1001	1979	p.P72R	missense_variant
1B	*FLT3*	13	2.9E+07	A	G	50.8%	1014	984	1998	-	splice_region_variant,intron_variant
1B	*KDR*	4	5.6E+07	T	A	100.0%	1972	0	1972	p.Q472H	missense_variant
1B	*TP53*	17	7579472	G	C	47.6%	950	1045	1995	p.P72R	missense_variant
2A	*FLT3*	13	2.9E+07	A	G	63.4%	1267	731	1998	-	splice_region_variant,intron_variant
2A	*KDR*	4	5.6E+07	T	A	54.3%	421	355	776	p.Q472H	missense_variant
2A	*TP53*	17	7579472	G	C	83.6%	1623	319	1942	p.P72R	missense_variant
2B	*FLT3*	13	2.9E+07	A	G	55.1%	1103	897	2000	-	splice_region_variant,intron_variant
2B	*KDR*	4	5.6E+07	T	A	50.4%	1003	986	1989	p.Q472H	missense_variant
2B	*TP53*	17	7579472	G	C	55.4%	1069	859	1928	p.P72R	missense_variant
3A	*FLT3*	13	2.9E+07	A	G	96.7%	1933	66	1999	-	splice_region_variant,intron_variant
3A	*KDR*	4	5.6E+07	T	A	39.9%	152	229	381	p.Q472H	missense_variant
3A	*TP53*	17	7579472	G	C	93.5%	1455	101	1556	p.P72R	missense_variant
3B	*FLT3*	13	2.9E+07	A	G	100.0%	1983	0	1983	-	splice_region_variant,intron_variant
3B	*KDR*	4	5.6E+07	T	A	50.6%	1010	986	1996	p.Q472H	missense_variant
3B	*TP53*	17	7579472	G	C	100.0%	1871	0	1871	p.P72R	missense_variant

4A	*FLT3*	13	2.9E+07	A	G	55.5%	1110	889	1999	-	splice_region_variant,intron_variant
4A	*KDR*	4	5.6E+07	T	A	100.0%	1976	0	1976	p.Q472H	missense_variant
4A	*TP53*	17	7579472	G	C	38.0%	757	1233	1990	p.P72R	missense_variant
4B	*FLT3*	13	2.9E+07	A	G	49.6%	991	1008	1999	-	splice_region_variant,intron_variant
4B	*KDR*	4	5.6E+07	T	A	98.9%	1972	22	1994	p.Q472H	missense_variant
4B	*TP53*	17	7579472	G	C	50.2%	1002	993	1995	p.P72R	missense_variant
5A	*FLT3*	13	2.9E+07	A	G	59.3%	1185	813	1998	-	splice_region_variant,intron_variant
10A	*TP53*	17	7577548	C	T	31.0%	619	1379	1998	p.G245S	missense_variant
5A	*TP53*	17	7579472	G	C	93.1%	1862	137	1999	p.P72R	missense_variant
5B	*FLT3*	13	2.9E+07	A	G	51.1%	1021	977	1998	-	splice_region_variant,intron_variant
10A	*APC*	5	1.1E+08	TC	T	18.4%	362	1606	1968	p.P1319fs	frameshift_variant,missense_variant,feature_truncation
11A	*FGFR1*	8	3.8E+07	GTCATCA	G	11.4%	218	1688	1906	p.D165_D166del	inframe_deletion
5B	*TP53*	17	7579472	G	C	93.6%	1869	127	1996	p.P72R	missense_variant
11A	*PTPN11*	12	1.1E+08	C	T	5.9%	119	1881	2000	p.Q510*	stop_gained
6A	*FLT3*	13	2.9E+07	A	G	100.0%	1984	0	1984	-	splice_region_variant,intron_variant
6A	*KIT*	4	5.6E+07	A	C	56.5%	1119	861	1980	p.M541L	missense_variant
6A	*TP53*	17	7579472	G	C	27.3%	544	1451	1995	p.P72R	missense_variant
13A	*TP53*	17	7578406	C	T	39.5%	784	1203	1987	p.R175H	missense_variant
6B	*FLT3*	13	2.9E+07	A	G	100.0%	1991	0	1991	-	splice_region_variant,intron_variant
6B	*KIT*	4	5.6E+07	A	C	50.8%	1009	976	1985	p.M541L	missense_variant
6B	*TP53*	17	7579472	G	C	52.5%	1006	909	1915	p.P72R	missense_variant
7A	*FLT3*	13	2.9E+07	A	G	43.1%	862	1136	1998	-	splice_region_variant,intron_variant

7A	*KDR*	4	5.6E+07	T	A	56.6%	639	489	1128	p.Q472H	missense_variant
7A	*MET*	7	1.2E+08	A	G	34.7%	686	1291	1977	p.N375S	missense_variant
7A	*TP53*	17	7579472	G	C	61.4%	270	170	440	p.P72R	missense_variant
13A	*APC*	5	1.1E+08	CT	C	27.6%	541	1416	1957	p.L1488fs	frameshift_variant,missense_variant,feature_truncation
13A	*KRAS*	12	2.5E+07	C	A	22.1%	418	1472	1890	p.G12V	missense_variant
14A	*TP53*	17	7577539	G	A	59.8%	1193	802	1995	p.R248W	missense_variant
14A	*APC*	5	1.1E+08	C	T	40.7%	811	1184	1995	p.Q1367*	stop_gained
15A	*TP53*	17	7578413	C	T	76.5%	861	239	1125	p.V173M	missense_variant
15A	*KRAS*	12	2.5E+07	C	A	44.9%	839	1029	1868	p.G12V	missense_variant
15A	*RET*	10	4.4E+07	C	T	5.2%	34	624	658	p.R635C	missense_variant
3A	*PTPN11*	12	1.1E+08	G	A	6.2%	91	1374	1465	p.R501K	missense_variant
7B	*FLT3*	13	2.9E+07	A	G	54.9%	1099	901	2000	-	splice_region_variant,intron_variant
7B	*KDR*	4	5.6E+07	T	A	44.8%	347	428	775	p.Q472H	missense_variant
7B	*MET*	7	1.2E+08	A	G	48.8%	964	1012	1976	p.N375S	missense_variant
17A	*TP53*	17	7577556	C	T	28.9%	569	1402	1971	p.C242Y	missense_variant
3A	*KDR*	4	5.6E+07	G	A	5.4%	33	575	608	-	splice_region_variant,intron_variant
18A	*TP53*	17	7578413	C	T	26.4%	523	1459	1982	p.V173M	missense_variant
18A	*APC*	5	1.1E+08	G	T	19.2%	384	1612	1996	p.E1353*	stop_gained
18A	*APC*	5	1.1E+08	G	T	15.8%	295	1576	1871	p.E1309*	stop_gained
18B	*TP53*	17	7578413	C	T	7.0%	139	1848	1987	p.V173M	missense_variant
18B	*APC*	5	1.1E+08	G	T	6.4%	127	1864	1991	p.E1353*	stop_gained
18B	*SMAD4*	18	4.9E+07	C	T	6.1%	122	1876	1998	p.A118V	missense_variant

18B	*APC*	5	1.1E+08	G	T	5.8%	109	1785	1894	p.E1309*	stop_gained
7B	*TP53*	17	7579472	G	C	62.7%	897	533	1430	p.P72R	missense_variant
8A	*STK11*	19	1223125	C	G	44.6%	891	1106	1997	p.F354L	missense_variant
19A	*TP53*	17	7577580	T	C	38.8%	759	1197	1956	p.Y234C	missense_variant
8A	*TP53*	17	7579472	G	C	94.3%	1660	101	1761	p.P72R	missense_variant
8B	*STK11*	19	1223125	C	G	47.5%	945	1045	1990	p.F354L	missense_variant
19A	*APC*	5	1.1E+08	C	G	27.4%	548	1449	1997	p.S1356*	stop_gained
19A	*TP53*	17	7578271	T	C	8.1%	161	1835	1996	p.H193R	missense_variant
8B	*TP53*	17	7579472	G	C	100.0%	1956	0	1956	p.P72R	missense_variant
9A	*FLT3*	13	2.9E+07	A	G	72.5%	1449	551	2000	-	splice_region_variant,intron_variant
9A	*KDR*	4	5.6E+07	T	A	70.1%	559	239	798	p.Q472H	missense_variant
19A	*FGFR3*	4	1808901	C	T	5.5%	44	750	794	p.P778L	missense_variant
9A	*STK11*	19	1223125	C	G	67.1%	638	313	951	p.F354L	missense_variant
1A	*SMAD4*	18	4.9E+07	G	A	6.9%	138	1858	1996	p.C127Y	missense_variant
20A	*TP53*	17	7578212	G	GA	11.2%	219	1728	1947	p.R213fs	frameshift_variant,feature_elongation
9A	*TP53*	17	7579472	G	C	71.8%	657	258	915	p.P72R	missense_variant
9B	*FLT3*	13	2.9E+07	A	G	100.0%	1990	0	1990	-	splice_region_variant,intron_variant
20B	*TP53*	17	7578212	G	GA	5.1%	98	1830	1928	p.R213fs	frameshift_variant,feature_elongation
21A	*TP53*	17	7578370	C	A	12.2%	245	1755	2000	p.?_splice	splice_donor_variant
21A	*APC*	5	1.1E+08	G	T	7.8%	156	1840	1996	p.E1513*	stop_gained
9B	*KDR*	4	5.6E+07	T	A	98.9%	1970	21	1991	p.Q472H	missense_variant
9B	*STK11*	19	1223125	C	G	39.1%	778	1212	1990	p.F354L	missense_variant

9B	*TP53*	17	7579472	G	C	50.2%	1000	994	1994	p.P72R	missense_variant
22A	*TP53*	17	7578406	C	T	24.6%	490	1501	1991	p.R175H	missense_variant
10A	*FLT3*	13	2.9E+07	A	G	47.4%	947	1052	1999	-	splice_region_variant,intron_variant
23A	*APC*	5	1.1E+08	C	CA	38.3%	757	1221	1978	p.M1383fs	frameshift_variant,feature_elongation
10A	*KDR*	4	5.6E+07	T	A	49.4%	984	1009	1993	p.Q472H	missense_variant
23A	*TP53*	17	7577570	C	T	37.6%	752	1246	1998	p.M237I	missense_variant
10A	*MLH1*	3	3.7E+07	T	A	54.5%	1084	905	1989	p.V384D	missense_variant
23A	*KRAS*	12	2.5E+07	C	T	21.7%	432	1563	1995	p.A146T	missense_variant
23B	*TP53*	17	7577570	C	T	6.9%	137	1862	1999	p.M237I	missense_variant
23B	*KRAS*	12	2.5E+07	C	T	5.1%	75	1387	1462	p.A146T	missense_variant
10A	*TP53*	17	7579472	G	C	70.6%	1343	558	1901	p.P72R	missense_variant
10B	*FLT3*	13	2.9E+07	A	G	53.8%	1076	923	1999	-	splice_region_variant,intron_variant
10B	*KDR*	4	5.6E+07	T	A	45.7%	909	1079	1988	p.Q472H	missense_variant
10B	*MLH1*	3	3.7E+07	T	A	47.0%	936	1054	1990	p.V384D	missense_variant
10B	*TP53*	17	7579472	G	C	54.6%	1032	858	1890	p.P72R	missense_variant
11A	*FLT3*	13	2.9E+07	A	G	100.0%	1986	0	1986	-	splice_region_variant,intron_variant
11A	*KDR*	4	5.6E+07	T	A	51.2%	1020	974	1994	p.Q472H	missense_variant
24A	*FLT3*	13	2.9E+07	T	C	49.3%	986	1013	1999	p.Y572C	missense_variant
11A	*TP53*	17	7579472	G	C	54.7%	1051	869	1920	p.P72R	missense_variant
11B	*FLT3*	13	2.9E+07	A	G	100.0%	1989	0	1989	-	splice_region_variant,intron_variant
24A	*MET*	7	1.2E+08	G	A	23.8%	474	1520	1994	p.D372N	missense_variant
11B	*KDR*	4	5.6E+07	T	A	48.6%	967	1023	1990	p.Q472H	missense_variant

24A	*ERBB4*	2	2.1E+08	C	A	17.6%	351	1638	1989	p.G340*	stop_gained
11B	*TP53*	17	7579472	G	C	53.6%	1025	889	1914	p.P72R	missense_variant
13A	*FLT3*	13	2.9E+07	A	G	100.0%	1989	0	1989	-	splice_region_variant,intron_variant
13B	*FLT3*	13	2.9E+07	A	G	100.0%	1980	0	1980	-	splice_region_variant,intron_variant
24A	*ATM*	11	1.1E+08	T	C	14.8%	296	1701	1997	p.V341A	missense_variant
14A	*FLT3*	13	2.9E+07	A	G	54.3%	1085	913	1998	-	splice_region_variant,intron_variant
24A	*PTEN*	10	9E+07	G	T	10.5%	209	1773	1982	p.E299*	stop_gained
14A	*KDR*	4	5.6E+07	T	A	33.7%	673	1323	1996	p.Q472H	missense_variant
14A	*TP53*	17	7579472	G	C	81.5%	1546	351	1897	p.P72R	missense_variant
24A	*TP53*	17	7579358	C	G	6.7%	97	1343	1440	p.R110P	missense_variant
24B	*FLT3*	13	2.9E+07	T	C	48.5%	969	1028	1997	p.Y572C	missense_variant
14B	*FLT3*	13	2.9E+07	A	G	50.7%	1014	985	1999	-	splice_region_variant,intron_variant
25A	*TP53*	17	7574003	G	A	39.7%	793	1206	1999	p.R342*	stop_gained
25A	*BRAF*	7	1.4E+08	A	T	15.4%	289	1586	1875	p.V600E	missense_variant
25B	*TP53*	17	7574003	G	A	22.7%	453	1546	1999	p.R342*	stop_gained
14B	*KDR*	4	5.6E+07	T	A	46.7%	929	1059	1988	p.Q472H	missense_variant
25B	*BRAF*	7	1.4E+08	A	T	11.4%	217	1690	1907	p.V600E	missense_variant
14B	*TP53*	17	7579472	G	C	57.3%	1098	819	1917	p.P72R	missense_variant
15A	*FLT3*	13	2.9E+07	A	G	100.0%	1981	0	1981	-	splice_region_variant,intron_variant
26A	*TP53*	17	7577559	G	A	40.0%	791	1188	1979	p.S241F	missense_variant
26A	*KRAS*	12	2.5E+07	T	A	17.8%	352	1625	1977	p.Q61L	missense_variant
27A	*KIT*	4	5.6E+07	A	T	51.2%	1017	969	1986	p.M651L	missense_variant

15A	*KDR*	4	5.6E+07	T	A	98.8%	1971	23	1994	p.Q472H	missense_variant
15A	*TP53*	17	7579472	G	C	17.5%	134	633	767	p.P72R	missense_variant
15B	*FLT3*	13	2.9E+07	A	G	100.0%	1985	0	1985	-	splice_region_variant,intron_variant
27A	*APC*	5	1.1E+08	G	T	23.3%	466	1530	1996	p.E1322*	stop_gained
15B	*KDR*	4	5.6E+07	T	A	100.0%	1984	0	1984	p.Q472H	missense_variant
15B	*TP53*	17	7579472	G	C	50.4%	982	968	1950	p.P72R	missense_variant
27A	*TP53*	17	7578460	A	T	22.5%	449	1543	1992	p.V157D	missense_variant
27A	*KRAS*	12	2.5E+07	C	A	13.7%	264	1669	1933	p.G12V	missense_variant
27B	*KIT*	4	5.6E+07	A	T	50.4%	1003	986	1989	p.M651L	missense_variant
28A	*MET*	7	1.2E+08	G	A	49.8%	986	992	1978	p.M840I	missense_variant
17A	*FLT3*	13	2.9E+07	A	G	99.0%	1979	19	1998	-	splice_region_variant,intron_variant
17A	*KDR*	4	5.6E+07	T	A	58.3%	1162	831	1993	p.Q472H	missense_variant
28A	*KRAS*	12	2.5E+07	C	T	18.3%	345	1541	1886	p.G12S	missense_variant
29A	*TP53*	17	7579377	G	A	23.6%	449	1452	1901	p.Q104*	stop_gained
17A	*MLH1*	3	3.7E+07	T	A	51.8%	1033	962	1995	p.V384D	missense_variant
29A	*FBXW7*	4	1.5E+08	G	A	12.1%	242	1756	1998	p.R505C	missense_variant
17A	*TP53*	17	7579472	G	C	66.5%	731	369	1100	p.P72R	missense_variant
17B	*FLT3*	13	2.9E+07	A	G	100.0%	1994	0	1994	-	splice_region_variant,intron_variant
17B	*KDR*	4	5.6E+07	T	A	47.9%	953	1037	1990	p.Q472H	missense_variant
29A	*APC*	5	1.1E+08	CT	C	10.3%	201	1750	1951	p.L1488fs	frameshift_variant,missense_variant,feature_truncation
17B	*MLH1*	3	3.7E+07	T	A	49.8%	991	998	1989	p.V384D	missense_variant
17B	*TP53*	17	7579472	G	C	50.8%	996	966	1962	p.P72R	missense_variant

29A	*APC*	5	1.1E+08	C	T	7.0%	139	1858	1997	p.R876*	stop_gained
29B	*TP53*	17	7579377	G	A	12.0%	235	1729	1964	p.Q104*	stop_gained
29B	*FBXW7*	4	1.5E+08	G	A	9.6%	191	1807	1998	p.R505C	missense_variant
18A	*FLT3*	13	2.9E+07	A	G	100.0%	1992	0	1992	-	splice_region_variant,intron_variant
29B	*APC*	5	1.1E+08	CT	C	7.9%	154	1801	1955	p.L1488fs	frameshift_variant,missense_variant,feature_truncation
2A	*TP53*	17	7577120	C	T	60.0%	1200	799	1999	p.R273H	missense_variant
18A	*KDR*	4	5.6E+07	T	A	50.1%	999	996	1995	p.Q472H	missense_variant
2A	*PTEN*	10	9E+07	C	T	5.7%	78	1284	1362	p.R233*	stop_gained
18A	*MET*	7	1.2E+08	A	G	43.4%	858	1118	1976	p.N375S	missense_variant
18A	*TP53*	17	7579472	G	C	37.8%	559	921	1480	p.P72R	missense_variant
18B	*FLT3*	13	2.9E+07	A	G	100.0%	1996	0	1996	-	splice_region_variant,intron_variant
2A	*PTEN*	10	9E+07	C	T	5.4%	31	546	577	p.P169L	missense_variant
18B	*KDR*	4	5.6E+07	T	A	51.5%	1026	966	1992	p.Q472H	missense_variant
18B	*MET*	7	1.2E+08	A	G	47.2%	931	1040	1971	p.N375S	missense_variant
18B	*TP53*	17	7579472	G	C	49.5%	963	981	1944	p.P72R	missense_variant
2A	*APC*	5	1.1E+08	G	A	5.2%	26	470	496	p.E1317K	missense_variant
30A	*ALK*	2	2.9E+07	C	T	38.1%	756	1229	1985	p.G1202E	missense_variant
19A	*FLT3*	13	2.9E+07	A	G	51.3%	1024	973	1997	-	splice_region_variant,intron_variant
30A	*TP53*	17	7577124	C	T	26.8%	535	1461	1996	p.V272M	missense_variant
19A	*KDR*	4	5.6E+07	T	A	53.5%	650	566	1216	p.Q472H	missense_variant
19A	*TP53*	17	7579472	G	C	91.5%	923	86	1009	p.P72R	missense_variant
19B	*FLT3*	13	2.9E+07	A	G	52.2%	1042	956	1998	-	splice_region_variant,intron_variant

19B	*KDR*	4	5.6E+07	T	A	49.0%	976	1017	1993	p.Q472H	missense_variant
19B	*TP53*	17	7579472	G	C	100.0%	1814	0	1814	p.P72R	missense_variant
20A	*FLT3*	13	2.9E+07	A	G	100.0%	1991	0	1991	-	splice_region_variant,intron_variant
30A	*APC*	5	1.1E+08	G	T	9.4%	180	1740	1920	p.E1306*	stop_gained
20A	*KDR*	4	5.6E+07	T	A	43.9%	876	1120	1996	p.Q472H	missense_variant
30A	*APC*	5	1.1E+08	C	T	9.1%	181	1818	1999	p.R876*	stop_gained
30B	*ALK*	2	2.9E+07	C	T	40.2%	800	1190	1990	p.G1202E	missense_variant
20A	*TP53*	17	7579472	G	C	100.0%	1482	0	1482	p.P72R	missense_variant
20B	*FLT3*	13	2.9E+07	A	G	100.0%	1980	0	1980	-	splice_region_variant,intron_variant
31A	*TP53*	17	7578190	T	C	11.4%	228	1767	1995	p.Y220C	missense_variant
20B	*KDR*	4	5.6E+07	T	A	49.0%	978	1018	1996	p.Q472H	missense_variant
31A	*KRAS*	12	2.5E+07	C	T	7.3%	48	614	662	p.A146T	missense_variant
20B	*TP53*	17	7579472	G	C	100.0%	1939	0	1939	p.P72R	missense_variant
21A	*FLT3*	13	2.9E+07	A	G	61.1%	1222	777	1999	-	splice_region_variant,intron_variant
31A	*CDKN2A*	9	2.2E+07	C	T	6.4%	35	502	546	p.G136D	missense_variant
31A	*KRAS*	12	2.5E+07	C	T	6.2%	41	615	656	p.R149K	missense_variant
31A	*ATM*	11	1.1E+08	G	A	5.3%	25	445	470	p.G327R	missense_variant
21A	*KDR*	4	5.6E+07	T	A	50.2%	1000	994	1994	p.Q472H	missense_variant
31A	*TP53*	17	7579478	G	A	5.3%	83	1470	1553	p.A70V	missense_variant
31A	*FBXW7*	4	1.5E+08	C	T	5.3%	28	497	525	p.E287K	missense_variant,splice_region_variant
21A	*MET*	7	1.2E+08	A	G	6.1%	121	1871	1992	p.N375S	missense_variant
31A	*FLT3*	13	2.9E+07	C	T	5.0%	36	690	726	p.S574N	missense_variant

3A	*TP53*	17	7578212	G	A	18.3%	353	1579	1932	p.R213*	stop_gained
3A	*FBXW7*	4	1.5E+08	G	A	8.1%	33	372	405	p.S282L	missense_variant
21A	*MLH1*	3	3.7E+07	T	A	42.3%	842	1147	1989	p.V384D	missense_variant
21A	*TP53*	17	7579472	G	C	43.1%	562	742	1304	p.P72R	missense_variant
3A	*ERBB4*	2	2.1E+08	G	A	7.6%	36	435	471	-	splice_region_variant,intron_variant
22A	*FLT3*	13	2.9E+07	A	G	38.4%	767	1232	1999	-	splice_region_variant,intron_variant
22A	*KDR*	4	5.6E+07	T	A	57.8%	1006	735	1741	p.Q472H	missense_variant
3A	*FBXW7*	4	1.5E+08	C	T	7.2%	29	375	404	p.?_splice	splice_donor_variant
22A	*TP53*	17	7579472	G	C	100.0%	1433	0	1433	p.P72R	missense_variant
22B	*FLT3*	13	2.9E+07	A	G	52.7%	1055	945	2000	-	splice_region_variant,intron_variant
22B	*KDR*	4	5.6E+07	T	A	50.2%	1000	993	1993	p.Q472H	missense_variant
22B	*TP53*	17	7579472	G	C	90.0%	1798	199	1997	p.P72R	missense_variant
23A	*FLT3*	13	2.9E+07	A	G	67.9%	1359	641	2000	-	splice_region_variant,intron_variant
23A	*KDR*	4	5.6E+07	T	A	36.7%	730	1260	1990	p.Q472H	missense_variant
3A	*FBXW7*	4	1.5E+08	G	A	6.9%	28	375	403	p.P274L	missense_variant
23A	*TP53*	17	7579472	G	C	100.0%	1094	0	1094	p.P72R	missense_variant
3A	*SMAD4*	18	4.9E+07	C	T	6.6%	78	1100	1178	p.P318S	missense_variant
23B	*FLT3*	13	2.9E+07	A	G	51.9%	1036	959	1995	-	splice_region_variant,intron_variant
23B	*KDR*	4	5.6E+07	T	A	48.2%	962	1033	1995	p.Q472H	missense_variant
23B	*TP53*	17	7579472	G	C	84.7%	1687	305	1992	p.P72R	missense_variant
24A	*FLT3*	13	2.9E+07	A	G	100.0%	1989	0	1989	-	splice_region_variant,intron_variant
24A	*KDR*	4	5.6E+07	T	A	51.5%	1027	969	1996	p.Q472H	missense_variant

24A	*TP53*	17	7579472	G	C	100.0%	1873	0	1873	p.P72R	missense_variant
3A	*KDR*	4	5.6E+07	C	T	6.6%	40	569	609	-	splice_region_variant,intron_variant
3A	*CTNNB1*	3	4.1E+07	C	T	6.3%	60	891	951	p.P44L	missense_variant
24B	*FLT3*	13	2.9E+07	A	G	100.0%	1991	0	1991	-	splice_region_variant,intron_variant
3A	*ERBB4*	2	2.1E+08	C	T	6.2%	77	1172	1249	p.G596D	missense_variant
24B	*KDR*	4	5.6E+07	T	A	51.4%	1024	970	1994	p.Q472H	missense_variant
3A	*ERBB4*	2	2.1E+08	C	T	6.1%	76	1161	1237	p.C617Y	missense_variant
3A	*ERBB4*	2	2.1E+08	C	T	6.0%	74	1166	1240	p.G586S	missense_variant
24B	*TP53*	17	7579472	G	C	100.0%	1971	0	1971	p.P72R	missense_variant
25A	*KDR*	4	5.6E+07	T	A	100.0%	1980	0	1980	p.Q472H	missense_variant
3A	*KRAS*	12	2.5E+07	C	T	6.0%	56	878	934	p.D30N	missense_variant
3A	*MET*	7	1.2E+08	C	T	5.9%	33	524	557	p.P822S	missense_variant
25A	*TP53*	17	7579472	G	C	71.7%	787	310	1097	p.P72R	missense_variant
25B	*KDR*	4	5.6E+07	T	A	100.0%	1977	0	1977	p.Q472H	missense_variant
25B	*TP53*	17	7579472	G	C	63.6%	1235	707	1942	p.P72R	missense_variant
3A	*ERBB4*	2	2.1E+08	G	A	5.8%	43	696	739	p.T360I	missense_variant
3A	*PTPN11*	12	1.1E+08	G	A	5.8%	59	953	1012	p.?_splice	splice_acceptor_variant
3A	*PIK3CA*	3	1.8E+08	G	A	5.8%	33	533	566	p.G320R	missense_variant
26A	*FLT3*	13	2.9E+07	A	G	100.0%	1988	0	1988	-	splice_region_variant,intron_variant
3A	*PTEN*	10	9E+07	C	T	5.5%	70	1206	1276	p.P246S	missense_variant
3A	*SMAD4*	18	4.9E+07	C	T	5.3%	62	1116	1178	p.P318L	missense_variant,splice_region_variant
26A	*TP53*	17	7579472	G	C	92.8%	1627	126	1753	p.P72R	missense_variant

3A	*ERBB4*	2	2.1E+08	C	T	5.2%	25	460	485	p.E314K	missense_variant
27A	*FLT3*	13	2.9E+07	A	G	54.3%	1083	912	1995	-	splice_region_variant,intron_variant
3A	*ERBB4*	2	2.1E+08	C	T	5.2%	25	460	485	p.C304Y	missense_variant
27A	*KDR*	4	5.6E+07	T	A	51.5%	1020	962	1982	p.Q472H	missense_variant
3A	*GNAQ*	9	8E+07	C	T	5.2%	66	1205	1271	p.E245K	missense_variant,splice_region_variant
3A	*PIK3CA*	3	1.8E+08	G	A	5.2%	35	633	668	p.D717N	missense_variant
3A	*EGFR*	7	5.5E+07	G	A	5.2%	70	1272	1342	p.R748K	missense_variant
3A	*CDKN2A*	9	2.2E+07	C	T	5.1%	64	1195	1259	p.D108N	missense_variant
3A	*PIK3CA*	3	1.8E+08	C	T	5.1%	27	498	525	p.T462I	missense_variant
27B	*FLT3*	13	2.9E+07	A	G	50.1%	1002	997	1999	-	splice_region_variant,intron_variant
3A	*KDR*	4	5.6E+07	C	T	5.1%	33	619	652	p.E1325K	missense_variant
27B	*KDR*	4	5.6E+07	T	A	51.5%	1025	967	1992	p.Q472H	missense_variant
3A	*FLT3*	13	2.9E+07	C	A	5.0%	42	806	848	p.G583V	missense_variant
28A	*FLT3*	13	2.9E+07	A	G	55.0%	1098	898	1996	-	splice_region_variant,intron_variant
3A	*SMAD4*	18	4.9E+07	G	A	5.0%	57	1079	1136	p.D120N	missense_variant
4A	*TP53*	17	7578406	C	T	32.0%	635	1349	1984	p.R175H	missense_variant
28A	*KDR*	4	5.6E+07	T	A	50.4%	1005	990	1995	p.Q472H	missense_variant
28A	*TP53*	17	7579472	G	C	52.5%	1004	908	1912	p.P72R	missense_variant
29A	*FLT3*	13	2.9E+07	A	G	100.0%	1993	0	1993	-	splice_region_variant,intron_variant
4A	*APC*	5	1.1E+08	A	T	24.9%	498	1499	1997	p.K1308*	stop_gained
4A	*FBXW7*	4	1.5E+08	G	A	11.9%	237	1762	1999	p.R505C	missense_variant
5A	*KRAS*	12	2.5E+07	C	T	54.3%	999	841	1840	p.G13D	missense_variant

5A	*TP53*	17	7577117	A	T	53.3%	1064	932	1996	p.V274D	missense_variant
29A	*KDR*	4	5.6E+07	T	A	52.4%	1045	951	1996	p.Q472H	missense_variant
29A	*TP53*	17	7579472	G	C	100.0%	1943	0	1943	p.P72R	missense_variant
5A	*SMAD4*	18	4.9E+07	A	T	38.3%	764	1230	1994	p.D360V	missense_variant
5A	*PIK3CA*	3	1.8E+08	G	A	29.2%	582	1413	1995	p.M1043I	missense_variant
6A	*FBXW7*	4	1.5E+08	G	A	33.2%	664	1333	1997	p.R505C	missense_variant
6A	*APC*	5	1.1E+08	C	CAGCT	22.7%	451	1534	1985	p.E1317fs	frameshift_variant,feature_elongation
6A	*KRAS*	12	2.5E+07	C	T	22.3%	423	1471	1894	p.G13D	missense_variant
7A	*TP53*	17	7577568	C	G	38.4%	762	1222	1984	p.C238S	missense_variant
7A	*STK11*	19	1220364	G	A	7.5%	28	344	372	-	splice_region_variant,intron_variant
7A	*GNA11*	19	3118938	G	A	6.5%	32	460	495	p.G208S	missense_variant
7A	*STK11*	19	1207066	G	A	5.2%	66	1206	1272	p.G52R	missense_variant
29B	*FLT3*	13	2.9E+07	A	G	100.0%	1986	0	1986	-	splice_region_variant,intron_variant
29B	*KDR*	4	5.6E+07	T	A	55.1%	1098	893	1991	p.Q472H	missense_variant
7B	*FBXW7*	4	1.5E+08	C	T	5.2%	34	616	650	p.G459E	missense_variant
29B	*TP53*	17	7579472	G	C	91.4%	1805	170	1975	p.P72R	missense_variant
8A	*TP53*	17	7579409	AGG	A	29.9%	489	1144	1637	p.L93fs	frameshift_variant,missense_variant,feature_truncation
30A	*FLT3*	13	2.9E+07	A	G	100.0%	1990	0	1990	-	splice_region_variant,intron_variant
30A	*KDR*	4	5.6E+07	T	A	100.0%	1979	0	1979	p.Q472H	missense_variant
8A	*KRAS*	12	2.5E+07	C	A	24.2%	467	1463	1930	p.G12V	missense_variant
8A	*APC*	5	1.1E+08	GA	G	16.4%	313	1592	1905	p.S1436fs	frameshift_variant,missense_variant,feature_truncation
30A	*TP53*	17	7579472	G	C	67.0%	1185	583	1768	p.P72R	missense_variant

30B	*FLT3*	13	2.9E+07	A	G	100.0%	1992	0	1992	-	splice_region_variant,intron_variant
9A	*TP53*	17	7578236	A	T	31.2%	620	1369	1989	p.Y205N	missense_variant
30B	*KDR*	4	5.6E+07	T	A	98.9%	1967	21	1988	p.Q472H	missense_variant
2A	*RET*	10	4.4E+07	G	A	5.4%	109	1891	2000	p.R886Q	missense_variant
9A	*KRAS*	12	2.5E+07	C	T	20.6%	253	975	1228	p.G12D	missense_variant
9A	*RB1*	13	4.9E+07	C	T	6.1%	28	428	456	p.R320*	stop_gained
9A	*STK11*	19	1220395	G	A	5.5%	36	621	657	p.G163D	missense_variant
30B	*TP53*	17	7579472	G	C	55.8%	1091	863	1954	p.P72R	missense_variant
31A	*FLT3*	13	2.9E+07	A	G	98.7%	1970	26	1996	-	splice_region_variant,intron_variant
31A	*KDR*	4	5.6E+07	T	A	56.3%	485	376	861	p.Q472H	missense_variant
9A	*CTNNB1*	3	4.1E+07	G	A	5.0%	63	1189	1252	p.G38S	missense_variant
31A	*TP53*	17	7579472	G	C	93.2%	1447	105	1552	p.P72R	missense_variant
31B	*FLT3*	13	2.9E+07	A	G	100.0%	1984	0	1984	-	splice_region_variant,intron_variant
31B	*KDR*	4	5.6E+07	T	A	50.5%	1006	988	1994	p.Q472H	missense_variant
31B	*TP53*	17	7579472	G	C	100.0%	1906	0	1906	p.P72R	missense_variant

A : specimens were collected by biopsy before nCRT
B : specimens were collected by surgery after nCRT
*indicated stop codon.

Supplementary Table S3. Synonymous variants of genes with more than 5% frequency identified in each library

Sample code	Chromosome coordinate					Variant information				Functional annotation	
	Gene symbol	Chr	Position	Ref	Var	Var freq	Var counts	Ref counts	Total depth	Protein change	consequence
10A	*FGFR3*	4	1807894	G	A	100.0%	1982	0	1982	p.T651T	synonymous_variant
10A	*PDGFRA*	4	5.5E+07	A	G	100.0%	1829	0	1829	p.P567P	synonymous_variant
10A	*APC*	5	1.1E+08	G	A	100.0%	1975	0	1975	p.T1493T	synonymous_variant
10A	*KIT*	4	5.6E+07	A	G	51.9%	1037	960	1997	p.K546K	synonymous_variant
10A	*RET*	10	4.4E+07	G	T	48.1%	957	1033	1990	p.L769L	synonymous_variant
10B	*FBXW7*	4	1.5E+08	T	C	5.0%	100	1898	1998	p.Q508Q	synonymous_variant
10B	*FGFR3*	4	1807894	G	A	100.0%	1985	0	1985	p.T651T	synonymous_variant
10B	*PDGFRA*	4	5.5E+07	A	G	100.0%	1862	0	1862	p.P567P	synonymous_variant
10B	*APC*	5	1.1E+08	G	A	99.9%	1962	0	1963	p.T1493T	synonymous_variant
10B	*KIT*	4	5.6E+07	A	G	50.2%	1005	995	2000	p.K546K	synonymous_variant
10B	*RET*	10	4.4E+07	G	T	49.5%	984	1004	1988	p.L769L	synonymous_variant
11A	*FGFR3*	4	1807894	G	A	100.0%	1975	0	1975	p.T651T	synonymous_variant
11A	*PDGFRA*	4	5.5E+07	A	G	100.0%	1838	0	1838	p.P567P	synonymous_variant
11A	*APC*	5	1.1E+08	G	A	100.0%	1969	0	1969	p.T1493T	synonymous_variant
11A	*EGFR*	7	5.5E+07	G	A	45.9%	656	773	1429	p.Q787Q	synonymous_variant
11A	*RET*	10	4.4E+07	G	T	100.0%	1970	0	1970	p.L769L	synonymous_variant
11B	*FGFR3*	4	1807894	G	A	100.0%	1980	0	1980	p.T651T	synonymous_variant
11B	*PDGFRA*	4	5.5E+07	A	G	100.0%	1867	0	1867	p.P567P	synonymous_variant

11B	*APC*	5	1.1E+08	G	A	100.0%	1959	0	1959	p.T1493T	synonymous_variant
11B	*EGFR*	7	5.5E+07	G	A	50.3%	1003	993	1996	p.Q787Q	synonymous_variant
11B	*RET*	10	4.4E+07	G	T	100.0%	1980	0	1980	p.L769L	synonymous_variant
13A	*KDR*	4	5.6E+07	C	T	43.7%	848	1091	1939	p.T1152T	synonymous_variant
13A	*FBXW7*	4	1.5E+08	T	C	5.3%	106	1892	1998	p.Q508Q	synonymous_variant
13A	*FGFR3*	4	1807894	G	A	100.0%	1982	0	1982	p.T651T	synonymous_variant
13A	*PDGFRA*	4	5.5E+07	A	G	100.0%	1859	0	1859	p.P567P	synonymous_variant
13A	*APC*	5	1.1E+08	G	A	52.5%	1039	940	1980	p.T1493T	synonymous_variant
13A	*EGFR*	7	5.5E+07	G	A	49.8%	994	1002	1996	p.Q787Q	synonymous_variant
13A	*RET*	10	4.4E+07	G	T	49.5%	985	1003	1988	p.L769L	synonymous_variant
13B	*KDR*	4	5.6E+07	C	T	49.0%	976	1016	1992	p.T1152T	synonymous_variant
13B	*FGFR3*	4	1807894	G	A	100.0%	1994	0	1994	p.T651T	synonymous_variant
13B	*PDGFRA*	4	5.5E+07	A	G	100.0%	1848	0	1848	p.P567P	synonymous_variant
13B	*APC*	5	1.1E+08	G	A	47.3%	922	1029	1951	p.T1493T	synonymous_variant
13B	*EGFR*	7	5.5E+07	G	A	48.9%	978	1020	1998	p.Q787Q	synonymous_variant
13B	*RET*	10	4.4E+07	G	T	51.8%	1027	956	1983	p.L769L	synonymous_variant
14A	*FGFR3*	4	1807894	G	A	100.0%	1929	0	1929	p.T651T	synonymous_variant
14A	*PDGFRA*	4	5.5E+07	A	G	100.0%	1825	0	1825	p.P567P	synonymous_variant
14A	*APC*	5	1.1E+08	G	A	41.0%	799	1151	1950	p.T1493T	synonymous_variant
14A	*KIT*	4	5.6E+07	A	G	32.5%	650	1348	1998	p.K546K	synonymous_variant
14A	*RET*	10	4.4E+07	G	T	58.6%	1168	824	1992	p.L769L	synonymous_variant
14A	*SMO*	7	1.3E+08	A	G	37.8%	756	1242	1998	p.E194E	synonymous_variant

14B	*FGFR3*	4	1807894	G	A	100.0%	1985	0	1985	p.T651T	synonymous_variant
14B	*PDGFRA*	4	5.5E+07	A	G	100.0%	1861	0	1861	p.P567P	synonymous_variant
14B	*APC*	5	1.1E+08	G	A	51.1%	1004	960	1964	p.T1493T	synonymous_variant
14B	*KIT*	4	5.6E+07	A	G	51.4%	1027	971	1998	p.K546K	synonymous_variant
14B	*RET*	10	4.4E+07	G	T	57.4%	1144	849	1993	p.L769L	synonymous_variant
14B	*SMO*	7	1.3E+08	A	G	52.3%	1045	953	1998	p.E194E	synonymous_variant
15A	*EGFR*	7	5.5E+07	G	A	49.8%	995	1004	1999	p.T725T	synonymous_variant
15A	*FGFR3*	4	1807894	G	A	100.0%	1555	0	1555	p.T651T	synonymous_variant
15A	*PDGFRA*	4	5.5E+07	A	G	100.0%	1812	0	1812	p.P567P	synonymous_variant
15A	*APC*	5	1.1E+08	G	A	78.4%	1530	422	1952	p.T1493T	synonymous_variant
15A	*EGFR*	7	5.5E+07	G	A	49.2%	587	606	1193	p.Q787Q	synonymous_variant
15A	*HRAS*	11	534242	A	G	46.5%	929	1069	1998	p.H27H	synonymous_variant
15A	*RET*	10	4.4E+07	G	T	24.3%	483	1504	1987	p.L769L	synonymous_variant
15B	*EGFR*	7	5.5E+07	G	A	50.5%	1010	990	2000	p.T725T	synonymous_variant
15B	*FBXW7*	4	1.5E+08	T	C	5.0%	100	1899	1999	p.Q508Q	synonymous_variant
15B	*FGFR3*	4	1807894	G	A	100.0%	1989	0	1989	p.T651T	synonymous_variant
15B	*PDGFRA*	4	5.5E+07	A	G	100.0%	1869	0	1869	p.P567P	synonymous_variant
15B	*APC*	5	1.1E+08	G	A	49.3%	967	995	1963	p.T1493T	synonymous_variant
15B	*EGFR*	7	5.5E+07	G	A	50.1%	819	816	1635	p.Q787Q	synonymous_variant
15B	*HRAS*	11	534242	A	G	48.9%	976	1020	1996	p.H27H	synonymous_variant
15B	*RET*	10	4.4E+07	G	T	48.4%	966	1028	1994	p.L769L	synonymous_variant
17A	*FBXW7*	4	1.5E+08	T	C	5.0%	99	1898	1997	p.Q508Q	synonymous_variant

17A	*FGFR3*	4	1807894	G	A	100.0%	1032	0	1032	p.T651T	synonymous_variant
17A	*PDGFRA*	4	5.5E+07	A	G	100.0%	1832	0	1832	p.P567P	synonymous_variant
17A	*APC*	5	1.1E+08	G	A	100.0%	1965	0	1965	p.T1493T	synonymous_variant
17A	*RET*	10	4.4E+07	G	T	57.4%	1143	848	1991	p.L769L	synonymous_variant
17A	*RET*	10	4.4E+07	C	G	54.6%	740	615	1355	p.S904S	synonymous_variant
17B	*FBXW7*	4	1.5E+08	T	C	5.9%	118	1877	1995	p.Q508Q	synonymous_variant
17B	*FGFR3*	4	1807894	G	A	100.0%	1985	0	1985	p.T651T	synonymous_variant
17B	*PDGFRA*	4	5.5E+07	A	G	100.0%	1804	0	1804	p.P567P	synonymous_variant
17B	*APC*	5	1.1E+08	G	A	100.0%	1974	0	1974	p.T1493T	synonymous_variant
17B	*RET*	10	4.4E+07	G	T	48.4%	961	1025	1986	p.L769L	synonymous_variant
17B	*RET*	10	4.4E+07	C	G	49.2%	979	1009	1988	p.S904S	synonymous_variant
18A	*FBXW7*	4	1.5E+08	T	C	5.2%	103	1895	1998	p.Q508Q	synonymous_variant
18A	*FGFR3*	4	1807894	G	A	100.0%	1986	0	1986	p.T651T	synonymous_variant
18A	*PDGFRA*	4	5.5E+07	A	G	99.9%	1861	1	1862	p.P567P	synonymous_variant
18A	*APC*	5	1.1E+08	G	A	100.0%	1974	0	1974	p.T1493T	synonymous_variant
18A	*MET*	7	1.2E+08	C	T	45.7%	911	1084	1995	p.S178S	synonymous_variant
18A	*RET*	10	4.4E+07	G	T	34.1%	678	1308	1986	p.L769L	synonymous_variant
18B	*FBXW7*	4	1.5E+08	T	C	5.5%	110	1888	1998	p.Q508Q	synonymous_variant
18B	*FGFR3*	4	1807894	G	A	100.0%	1978	0	1978	p.T651T	synonymous_variant
18B	*PDGFRA*	4	5.5E+07	A	G	100.0%	1870	0	1870	p.P567P	synonymous_variant
18B	*APC*	5	1.1E+08	G	A	100.0%	1972	0	1972	p.T1493T	synonymous_variant
18B	*MET*	7	1.2E+08	C	T	48.4%	965	1027	1992	p.S178S	synonymous_variant

18B	*RET*	10	4.4E+07	G	T	46.9%	935	1060	1995	p.L769L	synonymous_variant
19A	*FGFR3*	4	1807894	G	A	100.0%	1611	0	1611	p.T651T	synonymous_variant
19A	*PDGFRA*	4	5.5E+07	A	G	100.0%	994	0	994	p.P567P	synonymous_variant
19A	*APC*	5	1.1E+08	G	A	100.0%	1957	0	1957	p.T1493T	synonymous_variant
19A	*EGFR*	7	5.5E+07	G	A	49.9%	379	381	760	p.Q787Q	synonymous_variant
19A	*HRAS*	11	534242	A	G	30.6%	472	1070	1542	p.H27H	synonymous_variant
19A	*RET*	10	4.4E+07	G	T	45.5%	896	1075	1971	p.L769L	synonymous_variant
19A	*RET*	10	4.4E+07	C	G	42.0%	458	633	1091	p.S904S	synonymous_variant
19A	*SMO*	7	1.3E+08	A	G	45.4%	894	1075	1969	p.E194E	synonymous_variant
19B	*FGFR3*	4	1807894	G	A	100.0%	1440	0	1440	p.T651T	synonymous_variant
19B	*PDGFRA*	4	5.5E+07	A	G	100.0%	1863	0	1863	p.P567P	synonymous_variant
19B	*APC*	5	1.1E+08	G	A	100.0%	1978	0	1978	p.T1493T	synonymous_variant
19B	*EGFR*	7	5.5E+07	G	A	49.8%	656	662	1318	p.Q787Q	synonymous_variant
19B	*HRAS*	11	534242	A	G	47.8%	829	904	1733	p.H27H	synonymous_variant
19B	*RET*	10	4.4E+07	G	T	49.9%	991	994	1985	p.L769L	synonymous_variant
19B	*RET*	10	4.4E+07	C	G	49.0%	977	1015	1992	p.S904S	synonymous_variant
19B	*SMO*	7	1.3E+08	A	G	50.8%	1016	984	2000	p.E194E	synonymous_variant
1A	*FGFR3*	4	1807894	G	A	100.0%	1983	0	1983	p.T651T	synonymous_variant
1A	*IDH1*	2	2.1E+08	G	A	47.2%	942	1054	1996	p.G105G	synonymous_variant
1A	*PDGFRA*	4	5.5E+07	A	G	100.0%	1806	0	1806	p.P567P	synonymous_variant
1A	*APC*	5	1.1E+08	G	A	100.0%	1952	0	1952	p.T1493T	synonymous_variant
1A	*KIT*	4	5.6E+07	A	G	46.6%	928	1065	1993	p.K546K	synonymous_variant

1A	*SMO*	7	1.3E+08	A	G	49.5%	991	1009	2000	p.E194E	synonymous_variant
1B	*FGFR3*	4	1807894	G	A	100.0%	1991	0	1991	p.T651T	synonymous_variant
1B	*IDH1*	2	2.1E+08	G	A	50.6%	1007	982	1989	p.G105G	synonymous_variant
1B	*PDGFRA*	4	5.5E+07	A	G	100.0%	1795	0	1795	p.P567P	synonymous_variant
1B	*APC*	5	1.1E+08	G	A	100.0%	1969	0	1969	p.T1493T	synonymous_variant
1B	*KIT*	4	5.6E+07	A	G	50.9%	1017	981	1998	p.K546K	synonymous_variant
1B	*SMO*	7	1.3E+08	A	G	48.0%	959	1040	1999	p.E194E	synonymous_variant
20A	*FGFR3*	4	1807894	G	A	100.0%	1991	0	1991	p.T651T	synonymous_variant
20A	*PDGFRA*	4	5.5E+07	A	G	100.0%	1489	0	1489	p.P567P	synonymous_variant
20A	*APC*	5	1.1E+08	G	A	52.9%	1047	932	1979	p.T1493T	synonymous_variant
20B	*FGFR3*	4	1807894	G	A	100.0%	1986	0	1986	p.T651T	synonymous_variant
20B	*PDGFRA*	4	5.5E+07	A	G	100.0%	1835	0	1835	p.P567P	synonymous_variant
20B	*APC*	5	1.1E+08	G	A	48.5%	955	1013	1968	p.T1493T	synonymous_variant
21A	*FGFR3*	4	1807894	G	A	100.0%	1977	0	1977	p.T651T	synonymous_variant
21A	*PDGFRA*	4	5.5E+07	A	G	100.0%	1637	0	1637	p.P567P	synonymous_variant
21A	*APC*	5	1.1E+08	G	A	100.0%	1973	0	1973	p.T1493T	synonymous_variant
21A	*HRAS*	11	534242	A	G	42.3%	844	1153	1997	p.H27H	synonymous_variant
21A	*RET*	10	4.4E+07	G	T	8.2%	162	1821	1983	p.L769L	synonymous_variant
22A	*FGFR3*	4	1807894	G	A	100.0%	1984	0	1984	p.T651T	synonymous_variant
22A	*PDGFRA*	4	5.5E+07	A	G	100.0%	1763	0	1763	p.P567P	synonymous_variant
22A	*APC*	5	1.1E+08	G	A	60.4%	1196	783	1979	p.T1493T	synonymous_variant
22A	*HRAS*	11	534242	A	G	60.1%	1200	797	1997	p.H27H	synonymous_variant

22B	*FGFR3*	4	1807894	G	A	100.0%	1986	0	1986	p.T651T	synonymous_variant
22B	*PDGFRA*	4	5.5E+07	A	G	100.0%	1836	0	1836	p.P567P	synonymous_variant
22B	*APC*	5	1.1E+08	G	A	49.3%	966	995	1961	p.T1493T	synonymous_variant
22B	*HRAS*	11	534242	A	G	51.1%	1021	976	1997	p.H27H	synonymous_variant
23A	*FGFR3*	4	1807894	G	A	100.0%	1981	0	1981	p.T651T	synonymous_variant
23A	*PDGFRA*	4	5.5E+07	A	G	100.0%	1856	0	1856	p.P567P	synonymous_variant
23A	*APC*	5	1.1E+08	G	A	100.0%	1965	0	1965	p.T1493T	synonymous_variant
23A	*HRAS*	11	534242	A	G	98.4%	1965	32	1997	p.H27H	synonymous_variant
23A	*RET*	10	4.4E+07	G	T	61.5%	1218	762	1980	p.L769L	synonymous_variant
23B	*FGFR3*	4	1807894	G	A	100.0%	1975	0	1975	p.T651T	synonymous_variant
23B	*PDGFRA*	4	5.5E+07	A	G	100.0%	1599	0	1599	p.P567P	synonymous_variant
23B	*APC*	5	1.1E+08	G	A	100.0%	1958	0	1958	p.T1493T	synonymous_variant
23B	*HRAS*	11	534242	A	G	98.0%	1948	39	1987	p.H27H	synonymous_variant
23B	*RET*	10	4.4E+07	G	T	49.3%	980	1009	1989	p.L769L	synonymous_variant
24A	*FGFR3*	4	1807894	G	A	100.0%	1987	0	1987	p.T651T	synonymous_variant
24A	*PDGFRA*	4	5.5E+07	A	G	100.0%	1805	0	1805	p.P567P	synonymous_variant
24A	*APC*	5	1.1E+08	G	A	100.0%	1976	0	1976	p.T1493T	synonymous_variant
24A	*RET*	10	4.4E+07	G	T	52.2%	1037	949	1986	p.L769L	synonymous_variant
24A	*RET*	10	4.4E+07	C	G	50.4%	923	909	1832	p.S904S	synonymous_variant
24B	*FGFR3*	4	1807894	G	A	100.0%	1983	0	1983	p.T651T	synonymous_variant
24B	*PDGFRA*	4	5.5E+07	A	G	100.0%	1872	0	1872	p.P567P	synonymous_variant
24B	*APC*	5	1.1E+08	G	A	100.0%	1964	0	1964	p.T1493T	synonymous_variant

24B	*RET*	10	4.4E+07	G	T	51.5%	1023	962	1985	p.L769L	synonymous_variant
24B	*RET*	10	4.4E+07	C	G	54.2%	1080	913	1993	p.S904S	synonymous_variant
25A	*FGFR3*	4	1807894	G	A	99.0%	1002	10	1012	p.T651T	synonymous_variant
25A	*PDGFRA*	4	5.5E+07	A	G	100.0%	1837	0	1837	p.P567P	synonymous_variant
25A	*APC*	5	1.1E+08	G	A	55.5%	1089	873	1962	p.T1493T	synonymous_variant
25A	*EGFR*	7	5.5E+07	G	A	50.1%	331	330	661	p.Q787Q	synonymous_variant
25A	*HRAS*	11	534242	A	G	98.6%	1283	18	1301	p.H27H	synonymous_variant
25A	*PDGFRA*	4	5.5E+07	C	T	51.7%	1032	964	1996	p.V824V	synonymous_variant
25A	*RET*	10	4.4E+07	G	T	51.6%	1025	961	1986	p.L769L	synonymous_variant
25B	*FGFR3*	4	1807894	G	A	100.0%	1987	0	1987	p.T651T	synonymous_variant
25B	*PDGFRA*	4	5.5E+07	A	G	100.0%	1865	0	1865	p.P567P	synonymous_variant
25B	*APC*	5	1.1E+08	G	A	52.5%	1034	937	1971	p.T1493T	synonymous_variant
25B	*EGFR*	7	5.5E+07	G	A	51.5%	1028	970	1998	p.Q787Q	synonymous_variant
25B	*HRAS*	11	534242	A	G	100.0%	1980	0	1980	p.H27H	synonymous_variant
25B	*PDGFRA*	4	5.5E+07	C	T	47.8%	955	1043	1998	p.V824V	synonymous_variant
25B	*RET*	10	4.4E+07	G	T	48.3%	959	1028	1987	p.L769L	synonymous_variant
26A	*KRAS*	12	2.5E+07	T	G	17.7%	352	1635	1987	p.A59A	synonymous_variant
26A	*FGFR3*	4	1807894	G	A	100.0%	1363	0	1363	p.T651T	synonymous_variant
26A	*PDGFRA*	4	5.5E+07	A	G	100.0%	1871	0	1871	p.P567P	synonymous_variant
26A	*APC*	5	1.1E+08	G	A	62.1%	1220	745	1965	p.T1493T	synonymous_variant
26A	*HRAS*	11	534242	A	G	46.8%	880	1002	1882	p.H27H	synonymous_variant
26A	*RET*	10	4.4E+07	G	T	100.0%	1979	0	1979	p.L769L	synonymous_variant

26A	*RET*	10	4.4E+07	C	G	29.1%	579	1409	1988	p.S904S	synonymous_variant
26A	*SMO*	7	1.3E+08	A	G	98.6%	1972	28	2000	p.E194E	synonymous_variant
27A	*FGFR3*	4	1807894	G	A	100.0%	1428	0	1428	p.T651T	synonymous_variant
27A	*PDGFRA*	4	5.5E+07	A	G	100.0%	1853	0	1853	p.P567P	synonymous_variant
27A	*APC*	5	1.1E+08	G	A	100.0%	1961	0	1961	p.T1493T	synonymous_variant
27A	*RET*	10	4.4E+07	G	T	49.9%	990	993	1983	p.L769L	synonymous_variant
27A	*SMO*	7	1.3E+08	A	G	43.9%	877	1123	2000	p.E194E	synonymous_variant
27B	*FGFR3*	4	1807894	G	A	100.0%	1980	0	1980	p.T651T	synonymous_variant
27B	*PDGFRA*	4	5.5E+07	A	G	100.0%	1874	0	1874	p.P567P	synonymous_variant
27B	*APC*	5	1.1E+08	G	A	100.0%	1970	0	1970	p.T1493T	synonymous_variant
27B	*RET*	10	4.4E+07	G	T	47.8%	950	1038	1988	p.L769L	synonymous_variant
27B	*SMO*	7	1.3E+08	A	G	51.2%	1024	975	1999	p.E194E	synonymous_variant
28A	*FGFR3*	4	1807894	G	A	100.0%	1976	0	1976	p.T651T	synonymous_variant
28A	*PDGFRA*	4	5.5E+07	A	G	100.0%	1837	0	1837	p.P567P	synonymous_variant
28A	*APC*	5	1.1E+08	G	A	100.0%	1960	0	1960	p.T1493T	synonymous_variant
28A	*EGFR*	7	5.5E+07	G	A	54.2%	877	740	1617	p.Q787Q	synonymous_variant
28A	*RET*	10	4.4E+07	G	T	49.1%	973	1010	1983	p.L769L	synonymous_variant
28A	*RET*	10	4.4E+07	C	G	50.2%	960	951	1911	p.S904S	synonymous_variant
29A	*FGFR3*	4	1807894	G	A	100.0%	1830	0	1830	p.T651T	synonymous_variant
29A	*PDGFRA*	4	5.5E+07	A	G	100.0%	1820	0	1820	p.P567P	synonymous_variant
29A	*APC*	5	1.1E+08	G	A	100.0%	1953	0	1953	p.T1493T	synonymous_variant
29A	*EGFR*	7	5.5E+07	G	A	55.6%	850	679	1529	p.Q787Q	synonymous_variant

29A	*HRAS*	11	534242	A	G	49.1%	980	1017	1997	p.H27H	synonymous_variant
29A	*KIT*	4	5.6E+07	A	G	47.0%	940	1060	2000	p.K546K	synonymous_variant
29A	*PDGFRA*	4	5.5E+07	C	T	45.5%	908	1089	1997	p.V824V	synonymous_variant
29A	*RET*	10	4.4E+07	G	T	100.0%	1978	0	1978	p.L769L	synonymous_variant
29A	*RET*	10	4.4E+07	C	G	53.3%	1060	930	1990	p.S904S	synonymous_variant
29B	*FGFR3*	4	1807894	G	A	100.0%	1986	0	1986	p.T651T	synonymous_variant
29B	*PDGFRA*	4	5.5E+07	A	G	100.0%	1876	0	1876	p.P567P	synonymous_variant
29B	*APC*	5	1.1E+08	G	A	100.0%	1971	0	1971	p.T1493T	synonymous_variant
29B	*EGFR*	7	5.5E+07	G	A	47.6%	949	1046	1995	p.Q787Q	synonymous_variant
29B	*HRAS*	11	534242	A	G	50.7%	1010	984	1994	p.H27H	synonymous_variant
29B	*KIT*	4	5.6E+07	A	G	47.9%	958	1041	1999	p.K546K	synonymous_variant
29B	*PDGFRA*	4	5.5E+07	C	T	46.4%	927	1070	1997	p.V824V	synonymous_variant
29B	*RET*	10	4.4E+07	G	T	98.9%	1961	21	1982	p.L769L	synonymous_variant
29B	*RET*	10	4.4E+07	C	G	51.7%	1030	962	1992	p.S904S	synonymous_variant
2A	*ATM*	11	1.1E+08	G	A	6.0%	72	1135	1207	p.K1317K	synonymous_variant
2A	*FGFR3*	4	1806142	C	T	12.5%	250	1749	1999	p.I387I	synonymous_variant
2A	*JAK2*	9	5073748	G	A	5.5%	40	692	732	p.L609L	synonymous_variant
2A	*SMARCB1*	22	2.4E+07	C	T	5.1%	39	728	767	p.N380N	synonymous_variant
2A	*STK11*	19	1223122	C	G	77.9%	1536	436	1972	p.L353L	synonymous_variant
2A	*FGFR3*	4	1807894	G	A	100.0%	1988	0	1988	p.T651T	synonymous_variant
2A	*PDGFRA*	4	5.5E+07	A	G	100.0%	634	0	634	p.P567P	synonymous_variant
2A	*APC*	5	1.1E+08	G	A	100.0%	911	0	911	p.T1493T	synonymous_variant

2A	*PDGFRA*	4	5.5E+07	C	T	5.1%	90	1667	1757	p.V824V	synonymous_variant
2B	*FGFR3*	4	1806142	C	T	49.3%	985	1013	1998	p.I387I	synonymous_variant
2B	*STK11*	19	1223122	C	G	54.1%	1071	910	1981	p.L353L	synonymous_variant
2B	*FBXW7*	4	1.5E+08	T	C	5.0%	99	1899	1998	p.Q508Q	synonymous_variant
2B	*FGFR3*	4	1807894	G	A	100.0%	1984	0	1984	p.T651T	synonymous_variant
2B	*PDGFRA*	4	5.5E+07	A	G	100.0%	1838	0	1838	p.P567P	synonymous_variant
2B	*APC*	5	1.1E+08	G	A	99.9%	1964	0	1965	p.T1493T	synonymous_variant
30A	*FGFR1*	8	3.8E+07	C	T	34.8%	694	1299	1993	p.S158S	synonymous_variant
30A	*FGFR3*	4	1807894	G	A	100.0%	1796	0	1796	p.T651T	synonymous_variant
30A	*PDGFRA*	4	5.5E+07	A	G	100.0%	1860	0	1860	p.P567P	synonymous_variant
30A	*APC*	5	1.1E+08	G	A	100.0%	1961	0	1961	p.T1493T	synonymous_variant
30A	*RET*	10	4.4E+07	G	T	43.4%	861	1125	1986	p.L769L	synonymous_variant
30B	*FGFR1*	8	3.8E+07	C	T	45.5%	899	1078	1977	p.S158S	synonymous_variant
30B	*FGFR3*	4	1807894	G	A	100.0%	1977	0	1977	p.T651T	synonymous_variant
30B	*PDGFRA*	4	5.5E+07	A	G	100.0%	1833	0	1833	p.P567P	synonymous_variant
30B	*APC*	5	1.1E+08	G	A	100.0%	1955	0	1955	p.T1493T	synonymous_variant
30B	*RET*	10	4.4E+07	G	T	51.5%	1025	966	1991	p.L769L	synonymous_variant
31A	*FGFR3*	4	1807894	G	A	100.0%	1991	0	1991	p.T651T	synonymous_variant
31A	*PDGFRA*	4	5.5E+07	A	G	100.0%	716	0	716	p.P567P	synonymous_variant
31A	*APC*	5	1.1E+08	G	A	99.9%	1624	1	1625	p.T1493T	synonymous_variant
31A	*EGFR*	7	5.5E+07	G	A	100.0%	1513	0	1513	p.Q787Q	synonymous_variant
31A	*HRAS*	11	534242	A	G	50.7%	1012	985	1997	p.H27H	synonymous_variant

31A	*RET*	10	4.4E+07	G	T	57.3%	1135	845	1980	p.L769L	synonymous_variant
31B	*FGFR3*	4	1807894	G	A	100.0%	1986	0	1986	p.T651T	synonymous_variant
31B	*PDGFRA*	4	5.5E+07	A	G	100.0%	1811	0	1811	p.P567P	synonymous_variant
31B	*APC*	5	1.1E+08	G	A	100.0%	1944	0	1944	p.T1493T	synonymous_variant
31B	*EGFR*	7	5.5E+07	G	A	99.0%	1979	19	1998	p.Q787Q	synonymous_variant
31B	*HRAS*	11	534242	A	G	48.9%	975	1018	1993	p.H27H	synonymous_variant
31B	*RET*	10	4.4E+07	G	T	48.0%	956	1035	1991	p.L769L	synonymous_variant
3A	*ABL1*	9	1.3E+08	C	T	7.4%	79	987	1066	p.F317F	synonymous_variant
3A	*ATM*	11	1.1E+08	T	C	60.8%	1211	781	1992	p.A1931A	synonymous_variant
3A	*ATM*	11	1.1E+08	C	T	5.9%	55	882	937	p.F3049F	synonymous_variant
3A	*EGFR*	7	5.5E+07	G	A	5.3%	69	1196	1294	p.K757K	synonymous_variant
3A	*EGFR*	7	5.5E+07	C	T	6.6%	78	1109	1187	p.F795F	synonymous_variant
3A	*ERBB4*	2	2.1E+08	C	T	6.4%	47	693	740	p.V348V	synonymous_variant
3A	*FBXW7*	4	1.5E+08	G	A	5.7%	29	477	506	p.H455H	synonymous_variant
3A	*IDH2*	15	9.1E+07	G	A	6.4%	101	1479	1580	p.V147V	synonymous_variant
3A	*KDR*	4	5.6E+07	C	T	9.8%	92	849	941	p.K887K	synonymous_variant
3A	*KIT*	4	5.6E+07	G	A	5.9%	46	732	778	p.E53E	synonymous_variant
3A	*MET*	7	1.2E+08	C	T	6.6%	37	522	559	p.L827L	synonymous_variant
3A	*PDGFRA*	4	5.5E+07	C	T	5.4%	82	1445	1527	p.F678F	synonymous_variant
3A	*PTEN*	10	9E+07	G	A	5.1%	28	526	554	p.V175V	synonymous_variant
3A	*RET*	10	4.4E+07	C	T	6.7%	87	1213	1300	p.F644F	synonymous_variant
3A	*TP53*	17	7579423	G	A	7.4%	95	1182	1277	p.A88A	synonymous_variant

3A	*FGFR3*	4	1807894	G	A	100.0%	1991	0	1991	p.T651T	synonymous_variant
3A	*PDGFRA*	4	5.5E+07	A	G	100.0%	812	0	812	p.P567P	synonymous_variant
3A	*APC*	5	1.1E+08	G	A	54.8%	431	355	786	p.T1493T	synonymous_variant
3A	*EGFR*	7	5.5E+07	G	A	36.4%	432	756	1188	p.Q787Q	synonymous_variant
3A	*HRAS*	11	534242	A	G	48.1%	961	1038	1999	p.H27H	synonymous_variant
3A	*PDGFRA*	4	5.5E+07	C	T	8.6%	163	1728	1891	p.V824V	synonymous_variant
3A	*RET*	10	4.4E+07	G	T	100.0%	1980	0	1980	p.L769L	synonymous_variant
3A	*RET*	10	4.4E+07	C	G	100.0%	1343	0	1343	p.S904S	synonymous_variant
3A	*SMO*	7	1.3E+08	A	G	55.1%	1102	898	2000	p.E194E	synonymous_variant
3B	*ATM*	11	1.1E+08	T	C	50.4%	1002	986	1988	p.A1931A	synonymous_variant
3B	*FGFR3*	4	1807894	G	A	100.0%	1990	0	1990	p.T651T	synonymous_variant
3B	*PDGFRA*	4	5.5E+07	A	G	100.0%	1831	0	1831	p.P567P	synonymous_variant
3B	*APC*	5	1.1E+08	G	A	50.0%	978	979	1957	p.T1493T	synonymous_variant
3B	*EGFR*	7	5.5E+07	G	A	48.7%	973	1024	1997	p.Q787Q	synonymous_variant
3B	*HRAS*	11	534242	A	G	48.0%	957	1036	1993	p.H27H	synonymous_variant
3B	*RET*	10	4.4E+07	G	T	100.0%	1974	0	1974	p.L769L	synonymous_variant
3B	*RET*	10	4.4E+07	C	G	100.0%	1976	0	1976	p.S904S	synonymous_variant
3B	*SMO*	7	1.3E+08	A	G	47.2%	944	1056	2000	p.E194E	synonymous_variant
4A	*FGFR3*	4	1807894	G	A	100.0%	1982	0	1982	p.T651T	synonymous_variant
4A	*PDGFRA*	4	5.5E+07	A	G	100.0%	1840	0	1840	p.P567P	synonymous_variant
4A	*APC*	5	1.1E+08	G	A	37.6%	743	1232	1975	p.T1493T	synonymous_variant
4A	*RET*	10	4.4E+07	G	T	100.0%	1973	0	1973	p.L769L	synonymous_variant

4A	*RET*	10	4.4E+07	C	G	54.6%	1088	906	1994	p.S904S	synonymous_variant
4B	*FGFR3*	4	1807894	G	A	100.0%	1992	0	1992	p.T651T	synonymous_variant
4B	*PDGFRA*	4	5.5E+07	A	G	100.0%	1830	0	1830	p.P567P	synonymous_variant
4B	*APC*	5	1.1E+08	G	A	49.0%	962	1003	1965	p.T1493T	synonymous_variant
4B	*RET*	10	4.4E+07	G	T	100.0%	1981	0	1981	p.L769L	synonymous_variant
4B	*RET*	10	4.4E+07	C	G	43.5%	860	1115	1975	p.S904S	synonymous_variant
5A	*FBXW7*	4	1.5E+08	T	C	5.9%	118	1877	1995	p.Q508Q	synonymous_variant
5A	*FGFR3*	4	1807894	G	A	100.0%	1980	0	1980	p.T651T	synonymous_variant
5A	*PDGFRA*	4	5.5E+07	A	G	100.0%	1847	0	1847	p.P567P	synonymous_variant
5A	*APC*	5	1.1E+08	G	A	100.0%	1964	0	1964	p.T1493T	synonymous_variant
5A	*HRAS*	11	534242	A	G	49.8%	993	1002	1995	p.H27H	synonymous_variant
5A	*KIT*	4	5.6E+07	A	G	75.3%	1503	493	1996	p.K546K	synonymous_variant
5A	*RET*	10	4.4E+07	G	T	76.1%	1505	473	1978	p.L769L	synonymous_variant
5B	*FBXW7*	4	1.5E+08	T	C	5.5%	109	1889	1998	p.Q508Q	synonymous_variant
5B	*FGFR3*	4	1807894	G	A	100.0%	1990	0	1990	p.T651T	synonymous_variant
5B	*PDGFRA*	4	5.5E+07	A	G	100.0%	1847	0	1847	p.P567P	synonymous_variant
5B	*APC*	5	1.1E+08	G	A	100.0%	1966	0	1966	p.T1493T	synonymous_variant
5B	*HRAS*	11	534242	A	G	47.2%	942	1053	1995	p.H27H	synonymous_variant
5B	*KIT*	4	5.6E+07	A	G	48.4%	967	1030	1997	p.K546K	synonymous_variant
5B	*RET*	10	4.4E+07	G	T	47.7%	945	1038	1983	p.L769L	synonymous_variant
6A	*FGFR3*	4	1807894	G	A	100.0%	1970	0	1970	p.T651T	synonymous_variant
6A	*PDGFRA*	4	5.5E+07	A	G	100.0%	1857	0	1857	p.P567P	synonymous_variant

6A	*ALK*	2	2.9E+07	C	G	45.4%	908	1091	1999	p.A1200A	synonymous_variant
6A	*APC*	5	1.1E+08	G	A	100.0%	1965	0	1965	p.T1493T	synonymous_variant
6A	*PDGFRA*	4	5.5E+07	C	T	42.8%	855	1143	1998	p.V824V	synonymous_variant
6A	*RET*	10	4.4E+07	G	T	45.5%	904	1083	1987	p.L769L	synonymous_variant
6B	*FGFR3*	4	1807894	G	A	100.0%	1981	0	1981	p.T651T	synonymous_variant
6B	*PDGFRA*	4	5.5E+07	A	G	100.0%	1849	0	1849	p.P567P	synonymous_variant
6B	*ALK*	2	2.9E+07	C	G	43.8%	873	1118	1991	p.A1200A	synonymous_variant
6B	*APC*	5	1.1E+08	G	A	100.0%	1969	0	1969	p.T1493T	synonymous_variant
6B	*PDGFRA*	4	5.5E+07	C	T	49.6%	988	1004	1992	p.V824V	synonymous_variant
6B	*RET*	10	4.4E+07	G	T	51.8%	1032	962	1994	p.L769L	synonymous_variant
7A	*TP53*	17	7579465	G	A	6.5%	29	418	447	p.A74A	synonymous_variant
7A	*FGFR3*	4	1807894	G	A	100.0%	1165	0	1165	p.T651T	synonymous_variant
7A	*PDGFRA*	4	5.5E+07	A	G	100.0%	966	0	966	p.P567P	synonymous_variant
7A	*ALK*	2	2.9E+07	C	G	45.2%	482	584	1066	p.A1200A	synonymous_variant
7A	*APC*	5	1.1E+08	G	A	100.0%	1312	0	1312	p.T1493T	synonymous_variant
7A	*EGFR*	7	5.5E+07	G	A	100.0%	757	0	757	p.Q787Q	synonymous_variant
7A	*MET*	7	1.2E+08	C	T	42.7%	853	1144	1997	p.S178S	synonymous_variant
7B	*FGFR3*	4	1807894	G	A	100.0%	1981	0	1981	p.T651T	synonymous_variant
7B	*PDGFRA*	4	5.5E+07	A	G	99.9%	1856	1	1857	p.P567P	synonymous_variant
7B	*ALK*	2	2.9E+07	C	G	41.1%	818	1170	1988	p.A1200A	synonymous_variant
7B	*APC*	5	1.1E+08	G	A	100.0%	1961	0	1961	p.T1493T	synonymous_variant
7B	*EGFR*	7	5.5E+07	G	A	97.3%	1944	54	1998	p.Q787Q	synonymous_variant

7B	*MET*	7	1.2E+08	C	T	46.4%	926	1068	1994	p.S178S	synonymous_variant
8A	*FGFR3*	4	1807894	G	A	100.0%	1983	0	1983	p.T651T	synonymous_variant
8A	*PDGFRA*	4	5.5E+07	A	G	100.0%	1819	0	1819	p.P567P	synonymous_variant
8A	*APC*	5	1.1E+08	G	A	100.0%	1977	0	1977	p.T1493T	synonymous_variant
8A	*PDGFRA*	4	5.5E+07	C	T	44.9%	897	1103	2000	p.V824V	synonymous_variant
8A	*RET*	10	4.4E+07	G	T	100.0%	1984	0	1984	p.L769L	synonymous_variant
8A	*SMO*	7	1.3E+08	A	G	53.9%	1077	921	1998	p.E194E	synonymous_variant
8B	*FGFR3*	4	1807894	G	A	100.0%	1986	0	1986	p.T651T	synonymous_variant
8B	*PDGFRA*	4	5.5E+07	A	G	100.0%	1839	0	1839	p.P567P	synonymous_variant
8B	*APC*	5	1.1E+08	G	A	100.0%	1978	0	1978	p.T1493T	synonymous_variant
8B	*PDGFRA*	4	5.5E+07	C	T	49.9%	996	1001	1997	p.V824V	synonymous_variant
8B	*RET*	10	4.4E+07	G	T	100.0%	1972	0	1972	p.L769L	synonymous_variant
8B	*SMO*	7	1.3E+08	A	G	49.8%	995	1002	1997	p.E194E	synonymous_variant
9A	*STK11*	19	1220426	G	A	6.1%	40	617	657	p.V173V	synonymous_variant
9A	*FGFR3*	4	1807894	G	A	100.0%	1986	0	1986	p.T651T	synonymous_variant
9A	*PDGFRA*	4	5.5E+07	A	G	99.0%	880	9	889	p.P567P	synonymous_variant
9A	*APC*	5	1.1E+08	G	A	100.0%	1481	0	1481	p.T1493T	synonymous_variant
9A	*EGFR*	7	5.5E+07	G	A	42.1%	330	454	784	p.Q787Q	synonymous_variant
9A	*PDGFRA*	4	5.5E+07	C	T	13.1%	235	1565	1800	p.V824V	synonymous_variant
9A	*RET*	10	4.4E+07	G	T	13.3%	263	1719	1982	p.L769L	synonymous_variant
9A	*SMO*	7	1.3E+08	A	G	6.0%	120	1880	2000	p.E194E	synonymous_variant
9B	*FGFR3*	4	1807894	G	A	100.0%	1987	0	1987	p.T651T	synonymous_variant

9B	*PDGFRA*	4	5.5E+07	A	G	100.0%	1861	0	1861	p.P567P	synonymous_variant
9B	*APC*	5	1.1E+08	G	A	99.9%	1963	0	1964	p.T1493T	synonymous_variant
9B	*EGFR*	7	5.5E+07	G	A	53.8%	1073	922	1995	p.Q787Q	synonymous_variant

A : specimens were collected by biopsy before nCRT
B : specimens were collected by surgery after nCRT

Supplementary Table S4. Nonsynonymous variants of genes with 2%–5% frequency identified in each library

Sample code	Chromosome coordinate					Variant information				Functional annotation	
	Gene symbol	Chr	Position	Ref	Var	Var freq	Var counts	Ref counts	Total depth	Protein change	consequence
2A	*BRAF*	7	1.4E+08	G	A	4.4%	25	543	568	p.L584F	missense_variant
2A	*BRAF*	7	1.4E+08	C	T	4.2%	41	928	969	p.D449N	missense_variant
2A	*BRAF*	7	1.4E+08	G	A	2.3%	13	561	574	p.A598V	missense_variant
2A	*RET*	10	4.4E+07	G	A	4.6%	87	1796	1883	p.R897Q	missense_variant
2A	*PTEN*	10	9E+07	G	A	3.1%	40	1265	1305	p.W111*	stop_gained
2A	*PTEN*	10	9E+07	C	T	2.5%	33	1278	1311	p.R130*	stop_gained
2A	*PTEN*	10	9E+07	G	A	2.3%	13	557	570	p.G165E	missense_variant,splice_region_variant
2A	*PTEN*	10	9E+07	G	A	3.1%	18	558	576	p.S170N	missense_variant
2A	*PTEN*	10	9E+07	G	A	2.2%	24	1090	1114	p.?_splice	splice_acceptor_variant
2A	*PTEN*	10	9E+07	G	A	3.0%	42	1368	1410	p.G251D	missense_variant
2A	*PTEN*	10	9E+07	G	A	3.5%	37	1009	1046	p.A328T	missense_variant
2A	*ATM*	11	1.1E+08	C	T	3.5%	32	871	903	p.L409F	missense_variant
2A	*ATM*	11	1.1E+08	G	A	4.3%	12	266	278	p.E2884K	missense_variant
2A	*PTPN11*	12	1.1E+08	G	A	4.5%	55	1161	1216	p.V51I	missense_variant
2A	*PTPN11*	12	1.1E+08	G	A	2.1%	26	1198	1224	p.D61N	missense_variant
2A	*RB1*	13	4.9E+07	C	T	4.3%	21	467	488	p.T342I	missense_variant
2A	*RB1*	13	4.9E+07	G	A	3.6%	25	678	703	p.V366M	missense_variant
2A	*TP53*	17	7577018	C	T	2.1%	41	1959	2000	p.?_splice	splice_donor_variant

2A	*TP53*	17	7577030	C	T	2.4%	48	1951	1999	p.S303N	missense_variant
2A	*TP53*	17	7577099	C	T	2.6%	52	1947	1999	p.R280K	missense_variant
2A	*TP53*	17	7577114	C	T	2.8%	56	1943	1999	p.C275Y	missense_variant
2A	*MLH1*	3	3.7E+07	G	A	3.8%	63	1588	1651	p.M383I	missense_variant
2A	*TP53*	17	7579382	G	A	2.5%	48	1843	1891	p.T102I	missense_variant
2A	*TP53*	17	7579394	G	A	2.4%	47	1951	1998	p.P98L	missense_variant
2A	*SMAD4*	18	4.9E+07	C	T	2.5%	19	749	768	p.Q116*	stop_gained
2A	*SMAD4*	18	4.9E+07	G	A	3.7%	28	739	767	p.C127Y	missense_variant
2A	*SMAD4*	18	4.9E+07	G	A	4.8%	37	738	775	p.V335I	missense_variant
2A	*SMAD4*	18	4.9E+07	G	A	2.3%	20	868	888	p.A406T	missense_variant
2A	*SMAD4*	18	4.9E+07	G	A	3.7%	33	861	894	p.V409I	missense_variant
2A	*CTNNB1*	3	4.1E+07	C	T	2.5%	33	1277	1310	p.S33F	missense_variant
2A	*PIK3CA*	3	1.8E+08	G	A	3.1%	26	820	846	p.V344M	missense_variant
2A	*PIK3CA*	3	1.8E+08	G	A	4.5%	23	493	516	p.A415T	missense_variant
2A	*PIK3CA*	3	1.8E+08	G	A	2.2%	34	1529	1563	p.D538N	missense_variant
2A	*PIK3CA*	3	1.8E+08	G	A	3.7%	38	998	1036	p.M1043I	missense_variant
2A	*PIK3CA*	3	1.8E+08	C	T	2.7%	27	984	1011	p.H1047Y	missense_variant
2A	*PIK3CA*	3	1.8E+08	G	A	2.3%	23	964	987	p.G1050S	missense_variant
2A	*KIT*	4	5.6E+07	C	T	2.4%	25	1009	1034	p.P573L	missense_variant
2A	*KIT*	4	5.6E+07	C	T	3.6%	37	1000	1037	p.P577L	missense_variant
2A	*KIT*	4	5.6E+07	G	A	3.5%	31	849	880	p.G648D	missense_variant
2A	*KDR*	4	5.6E+07	G	A	3.5%	27	750	777	p.A473V	missense_variant

2A	*APC*	5	1.1E+08	G	A	3.5%	42	1150	1192	p.C1098Y	missense_variant
2A	*APC*	5	1.1E+08	G	A	4.2%	20	458	478	p.E1286K	missense_variant
2A	*APC*	5	1.1E+08	C	T	2.5%	25	982	1007	p.Q1367*	stop_gained
2A	*APC*	5	1.1E+08	C	T	2.4%	23	942	965	p.Q1378*	stop_gained
2A	*APC*	5	1.1E+08	G	A	4.1%	33	773	806	p.V1452I	missense_variant
2A	*EGFR*	7	5.5E+07	C	T	2.5%	50	1945	1995	p.A289V	missense_variant
2A	*EGFR*	7	5.5E+07	G	A	2.2%	44	1956	2000	p.G810D	missense_variant
2A	*ERBB4*	2	2.1E+08	C	T	3.8%	49	1246	1295	p.D585N	missense_variant
2B	*TP53*	17	7577120	C	T	4.4%	87	1912	1999	p.R273H	missense_variant
30A	*CDKN2A*	9	2.2E+07	G	A	3.7%	15	395	410	p.T137I	missense_variant
30B	*SMARCB1*	22	2.4E+07	C	A	4.6%	65	1346	1411	p.T72K	missense_variant
31A	*BRAF*	7	1.4E+08	C	T	4.8%	34	675	709	p.G593D	missense_variant
31A	*PTEN*	10	9E+07	G	A	2.1%	42	1958	2000	p.G127R	missense_variant
31A	*PTEN*	10	9E+07	G	A	2.0%	23	1099	1122	p.S170N	missense_variant
31A	*PTEN*	10	9E+07	C	T	2.2%	36	1576	1612	p.Q214*	stop_gained
31A	*PTPN11*	12	1.1E+08	G	A	2.6%	49	1869	1918	p.D61N	missense_variant
31A	*PTPN11*	12	1.1E+08	G	A	4.4%	85	1829	1914	p.G68R	missense_variant
31A	*FLT3*	13	2.9E+07	C	T	4.5%	31	665	696	p.W603*	stop_gained
31A	*IDH2*	15	9.1E+07	G	A	2.1%	41	1912	1953	p.R140W	missense_variant
31A	*TP53*	17	7579422	G	A	3.1%	48	1480	1528	p.P89S	missense_variant
31A	*ERBB2*	17	3.8E+07	G	A	2.5%	49	1911	1960	p.V842I	missense_variant
31A	*SMAD4*	18	4.9E+07	C	T	2.0%	20	993	1013	p.R135*	stop_gained

31A	*SMAD4*	18	4.9E+07	G	A	2.8%	48	1650	1698	p.R361H	missense_variant
31A	*SMAD4*	18	4.9E+07	G	A	2.4%	35	1432	1467	p.A406T	missense_variant
31A	*SMAD4*	18	4.9E+07	C	T	2.2%	43	1955	1998	p.R531W	missense_variant
31A	*PDGFRA*	4	5.5E+07	G	A	3.9%	62	1508	1570	p.E704K	missense_variant
31A	*KIT*	4	5.6E+07	C	T	3.6%	46	1225	1271	p.H580Y	missense_variant
31A	*KDR*	4	5.6E+07	G	A	4.5%	38	801	839	p.S475L	missense_variant
31A	*KDR*	4	5.6E+07	C	T	3.9%	36	892	928	p.W260*	stop_gained
31A	*KDR*	4	5.6E+07	C	T	3.8%	35	891	926	p.A248T	missense_variant
31A	*APC*	5	1.1E+08	G	T	4.7%	81	1632	1713	p.G1312*	stop_gained
31A	*APC*	5	1.1E+08	C	T	3.6%	12	320	332	p.T1556I	missense_variant
31A	*EGFR*	7	5.5E+07	C	T	2.1%	41	1959	2000	p.L730F	missense_variant
31A	*EGFR*	7	5.5E+07	G	A	3.0%	55	1770	1825	p.E746K	missense_variant
31A	*EGFR*	7	5.5E+07	G	A	2.1%	42	1958	2000	p.D761N	missense_variant,splice_region_variant
31A	*IDH1*	2	2.1E+08	G	A	2.2%	43	1956	1999	p.R132C	missense_variant
31A	*FGFR1*	8	3.8E+07	G	A	4.9%	32	618	650	p.S169L	missense_variant
31A	*CDKN2A*	9	2.2E+07	G	A	3.8%	27	676	703	p.R131C	missense_variant
31A	*CDKN2A*	9	2.2E+07	C	T	3.7%	28	726	754	p.V126I	missense_variant
31A	*CDKN2A*	9	2.2E+07	G	A	4.8%	36	716	752	p.A109V	missense_variant
31A	*CDKN2A*	9	2.2E+07	G	A	2.8%	21	716	737	p.A102V	missense_variant
31A	*CDKN2A*	9	2.2E+07	C	T	3.8%	28	710	738	p.A102T	missense_variant
31B	*KRAS*	12	2.5E+07	C	T	3.2%	64	1933	1997	p.A146T	missense_variant
3A	*ERBB4*	2	2.1E+08	C	T	3.9%	19	466	485	p.C308Y	missense_variant

3A	*FGFR1*	8	3.8E+07	G	A	3.8%	73	1857	1930	p.Q289*	stop_gained
3A	*FGFR1*	8	3.8E+07	C	T	4.8%	23	455	478	-	splice_region_variant,intron_variant
3A	*FGFR1*	8	3.8E+07	C	T	4.2%	23	527	550	p.E170K	missense_variant
3A	*FGFR1*	8	3.8E+07	C	T	4.0%	30	724	754	p.D164N	missense_variant
3A	*JAK2*	9	5073731	C	G	3.8%	13	326	339	p.L604V	missense_variant
3A	*CDKN2A*	9	2.2E+07	C	T	4.0%	51	1217	1268	p.G122S	missense_variant
3A	*CDKN2A*	9	2.2E+07	C	T	3.7%	47	1213	1260	p.A109T	missense_variant
3A	*CDKN2A*	9	2.2E+07	C	T	3.7%	46	1210	1256	p.R107H	missense_variant
3A	*CDKN2A*	9	2.2E+07	G	A	4.6%	57	1190	1247	p.R103W	missense_variant
3A	*ABL1*	9	1.3E+08	G	A	3.6%	41	1096	1137	p.V338M	missense_variant
3A	*ABL1*	9	1.3E+08	G	A	3.6%	73	1927	2000	p.W405*	stop_gained
3A	*NOTCH1*	9	1.4E+08	C	T	3.6%	72	1925	1997	p.G2447S	missense_variant
3A	*RET*	10	4.4E+07	C	T	4.1%	58	1372	1430	p.P628L	missense_variant
3A	*RET*	10	4.4E+07	G	A	4.8%	65	1278	1343	p.G885R	missense_variant
3A	*PTEN*	10	9E+07	G	A	4.9%	14	272	286	p.?_splice	splice_acceptor_variant
3A	*PTEN*	10	9E+07	G	A	2.2%	29	1267	1296	p.W111*	stop_gained
3A	*PTEN*	10	9E+07	T	C	3.5%	46	1254	1300	p.L112P	missense_variant
3A	*PTEN*	10	9E+07	G	A	2.0%	26	1283	1309	p.V119I	missense_variant
3A	*PTEN*	10	9E+07	G	A	2.0%	26	1291	1317	p.G127E	missense_variant
3A	*PTEN*	10	9E+07	G	A	2.2%	29	1271	1300	p.R130Q	missense_variant
3A	*PTEN*	10	9E+07	G	A	3.2%	33	1002	1035	p.?_splice	splice_acceptor_variant
3A	*PTEN*	10	9E+07	G	A	2.1%	41	1955	1996	p.E291K	missense_variant

3A	*ATM*	11	1.1E+08	G	A	3.5%	17	462	479	p.G335E	missense_variant
3A	*ATM*	11	1.1E+08	C	T	4.0%	57	1373	1430	p.P1680S	missense_variant
3A	*ATM*	11	1.1E+08	C	T	4.7%	33	669	702	-	splice_region_variant,intron_variant
3A	*ATM*	11	1.1E+08	C	T	4.0%	28	672	700	p.Q2730*	stop_gained
3A	*ATM*	11	1.1E+08	C	T	2.8%	26	908	934	p.R3047*	stop_gained
3A	*PTPN11*	12	1.1E+08	G	A	3.0%	32	1023	1055	p.D61N	missense_variant
3A	*PTPN11*	12	1.1E+08	G	A	2.4%	25	1025	1050	p.E69K	missense_variant
3A	*PTPN11*	12	1.1E+08	G	A	3.2%	34	1014	1048	p.A72T	missense_variant
3A	*FLT3*	13	2.9E+07	C	T	3.6%	29	770	799	p.R607K	missense_variant
3A	*FLT3*	13	2.9E+07	C	T	4.2%	35	805	840	p.E604K	missense_variant
3A	*FLT3*	13	2.9E+07	C	T	3.7%	32	842	874	p.V592I	missense_variant
3A	*RB1*	13	4.9E+07	C	T	4.1%	35	815	850	p.Q685*	stop_gained
3A	*RB1*	13	4.9E+07	C	T	3.8%	32	818	850	p.H686Y	missense_variant
3A	*AKT1*	14	1.1E+08	G	A	3.9%	51	1242	1293	p.Q47*	stop_gained
3A	*CDH1*	16	6.9E+07	G	A	4.3%	61	1372	1433	p.?_splice	splice_acceptor_variant
3A	*CDH1*	16	6.9E+07	C	T	4.6%	66	1379	1445	p.P339L	missense_variant
3A	*TP53*	17	7577019	C	T	3.9%	53	1290	1343	p.A307T	missense_variant,splice_region_variant
3A	*TP53*	17	7577108	C	T	3.0%	42	1345	1387	p.C277Y	missense_variant
3A	*TP53*	17	7577114	C	T	3.6%	50	1337	1387	p.C275Y	missense_variant
3A	*TP53*	17	7577124	C	T	2.8%	39	1345	1384	p.V272M	missense_variant
3A	*VHL*	3	1E+07	C	T	3.7%	73	1926	1999	p.L140F	missense_variant
3A	*TP53*	17	7577532	G	A	2.3%	46	1953	1999	p.P250L	missense_variant

3A	*TP53*	17	7577552	C	T	3.9%	78	1920	1998	p.M243I	missense_variant
3A	*TP53*	17	7578190	T	C	2.7%	53	1945	1998	p.Y220C	missense_variant
3A	*TP53*	17	7578423	C	T	2.3%	45	1950	1995	p.M169I	missense_variant
3A	*TP53*	17	7578450	C	T	2.5%	49	1950	1999	p.M160I	missense_variant
3A	*TP53*	17	7578457	C	T	2.4%	48	1940	1988	p.R158H	missense_variant
3A	*TP53*	17	7578476	G	A	2.0%	40	1958	1998	p.P152S	missense_variant
3A	*TP53*	17	7579382	G	A	2.2%	30	1313	1343	p.T102I	missense_variant
3A	*TP53*	17	7579391	G	A	2.0%	28	1396	1424	p.S99F	missense_variant
3A	*TP53*	17	7579395	G	A	2.4%	35	1412	1447	p.P98S	missense_variant
3A	*ERBB2*	17	3.8E+07	G	A	4.1%	81	1919	2000	p.E876K	missense_variant
3A	*SMAD4*	18	4.9E+07	G	A	4.1%	47	1092	1139	p.C115Y	missense_variant
3A	*SMAD4*	18	4.9E+07	G	A	4.3%	47	1049	1096	p.V136I	missense_variant
3A	*SMAD4*	18	4.9E+07	C	T	3.9%	78	1921	1999	p.P198S	missense_variant
3A	*SMAD4*	18	4.9E+07	C	T	3.7%	33	864	897	p.H261Y	missense_variant
3A	*SMAD4*	18	4.9E+07	C	T	4.2%	49	1117	1166	p.P313S	missense_variant
3A	*SMAD4*	18	4.9E+07	G	A	4.0%	27	645	672	p.V341I	missense_variant
3A	*SMAD4*	18	4.9E+07	G	A	2.2%	16	723	739	p.G386D	missense_variant
3A	*SMAD4*	18	4.9E+07	G	A	4.1%	30	710	740	p.E394K	missense_variant
3A	*SMAD4*	18	4.9E+07	G	A	4.3%	32	712	744	p.D396N	missense_variant
3A	*SMAD4*	18	4.9E+07	G	A	4.5%	34	721	755	p.D415N	missense_variant
3A	*SMAD4*	18	4.9E+07	G	A	3.8%	58	1451	1509	p.G508S	missense_variant
3A	*SMAD4*	18	4.9E+07	C	T	2.9%	42	1408	1450	p.R531W	missense_variant

3A	*SMARCB1*	22	2.4E+07	G	A	3.9%	78	1921	1999	p.D170N	missense_variant
3A	*SMARCB1*	22	2.4E+07	G	A	4.6%	42	874	916	p.R376K	missense_variant
3A	*SMARCB1*	22	2.4E+07	G	A	3.7%	31	817	848	p.A379T	missense_variant
3A	*CTNNB1*	3	4.1E+07	G	A	2.5%	25	986	1011	p.G38D	missense_variant
3A	*CTNNB1*	3	4.1E+07	G	A	3.0%	27	868	895	p.S47N	missense_variant
3A	*PIK3CA*	3	1.8E+08	G	A	4.3%	10	225	235	p.R108H	missense_variant
3A	*PIK3CA*	3	1.8E+08	G	T	4.4%	24	523	547	p.M318I	missense_variant
3A	*PIK3CA*	3	1.8E+08	C	T	3.9%	22	545	567	p.S323F	missense_variant
3A	*PIK3CA*	3	1.8E+08	G	A	4.2%	24	554	578	p.W328*	stop_gained
3A	*PIK3CA*	3	1.8E+08	G	A	3.8%	22	558	580	p.R335K	missense_variant
3A	*PIK3CA*	3	1.8E+08	C	T	4.2%	24	554	578	p.R349*	stop_gained
3A	*PIK3CA*	3	1.8E+08	G	A	2.6%	42	1597	1639	p.E547K	missense_variant
3A	*PIK3CA*	3	1.8E+08	G	A	4.3%	41	903	944	p.D1018N	missense_variant
3A	*PIK3CA*	3	1.8E+08	G	A	3.1%	27	841	868	p.G1049D	missense_variant
3A	*PIK3CA*	3	1.8E+08	G	A	2.7%	23	842	865	p.G1050D	missense_variant
3A	*PDGFRA*	4	5.5E+07	C	T	3.8%	40	1020	1060	p.P553L	missense_variant
3A	*PDGFRA*	4	5.5E+07	G	A	4.1%	62	1455	1517	p.E675K	missense_variant
3A	*PDGFRA*	4	5.5E+07	G	A	3.8%	72	1805	1877	p.V824I	missense_variant
3A	*KIT*	4	5.6E+07	G	A	4.2%	33	746	779	p.R55K	missense_variant
3A	*KIT*	4	5.6E+07	G	A	3.9%	44	1092	1136	p.G498S	missense_variant
3A	*KIT*	4	5.6E+07	G	A	4.7%	32	656	688	p.V559I	missense_variant
3A	*KIT*	4	5.6E+07	G	A	4.7%	32	643	675	p.R586K	missense_variant

3A	*KDR*	4	5.6E+07	C	T	4.7%	31	632	663	p.G1348R	missense_variant
3A	*KDR*	4	5.6E+07	G	A	4.1%	38	898	936	p.H891Y	missense_variant
3A	*KDR*	4	5.6E+07	G	A	4.8%	29	581	610	p.S264F	missense_variant
3A	*KDR*	4	5.6E+07	G	A	3.8%	23	587	610	p.P263L	missense_variant
3A	*FBXW7*	4	1.5E+08	C	T	4.6%	23	482	505	p.E452K	missense_variant
3A	*FBXW7*	4	1.5E+08	C	T	3.8%	19	486	505	p.D440N	missense_variant
3A	*FBXW7*	4	1.5E+08	C	T	4.0%	20	484	504	p.G437E	missense_variant
3A	*FBXW7*	4	1.5E+08	C	T	3.8%	19	484	503	p.S436N	missense_variant
3A	*APC*	5	1.1E+08	C	T	3.5%	53	1440	1493	p.P1091L	missense_variant
3A	*APC*	5	1.1E+08	G	A	4.3%	22	489	511	p.E1295K	missense_variant
3A	*APC*	5	1.1E+08	G	A	4.4%	22	476	498	p.E1306K	missense_variant
3A	*APC*	5	1.1E+08	C	T	4.0%	36	863	899	p.S1346L	missense_variant
3A	*APC*	5	1.1E+08	C	T	4.3%	39	859	898	p.S1360F	missense_variant
3A	*APC*	5	1.1E+08	C	T	2.9%	26	860	886	p.Q1378*	stop_gained
3A	*APC*	5	1.1E+08	G	A	4.2%	37	852	889	p.E1379K	missense_variant
3A	*APC*	5	1.1E+08	C	T	3.5%	31	851	882	p.T1380I	missense_variant
3A	*APC*	5	1.1E+08	C	T	3.5%	30	821	851	p.L1382F	missense_variant
3A	*APC*	5	1.1E+08	G	A	2.6%	31	1171	1202	p.E1451K	missense_variant
3A	*ALK*	2	2.9E+07	C	T	2.1%	39	1776	1815	p.R1275Q	missense_variant
3A	*APC*	5	1.1E+08	C	T	3.9%	32	792	824	p.S1501F	missense_variant
3A	*APC*	5	1.1E+08	G	A	3.6%	30	799	829	p.S1505N	missense_variant
3A	*APC*	5	1.1E+08	C	T	3.5%	29	790	819	p.Q1517*	stop_gained

3A	*ALK*	2	2.9E+07	G	A	4.1%	56	1299	1355	p.P1191L	missense_variant
3A	*ALK*	2	2.9E+07	C	T	3.5%	48	1313	1361	p.G1184R	missense_variant
3A	*ALK*	2	2.9E+07	C	T	4.2%	57	1303	1360	p.V1180I	missense_variant
3A	*EGFR*	7	5.5E+07	C	T	4.5%	74	1569	1643	p.P281L	missense_variant
3A	*EGFR*	7	5.5E+07	G	A	2.1%	29	1355	1384	p.G735S	missense_variant
3A	*EGFR*	7	5.5E+07	C	T	2.2%	29	1196	1294	p.T751I	missense_variant
3A	*EGFR*	7	5.5E+07	G	A	3.0%	42	1354	1396	p.D761N	missense_variant,splice_region_variant
3A	*EGFR*	7	5.5E+07	C	T	2.0%	23	1148	1171	p.A767V	missense_variant
3A	*EGFR*	7	5.5E+07	G	A	4.8%	57	1137	1194	p.G779S	missense_variant
3A	*EGFR*	7	5.5E+07	C	T	4.5%	54	1136	1190	p.P794S	missense_variant
3A	*EGFR*	7	5.5E+07	G	A	4.1%	75	1745	1820	p.G810S	missense_variant
3A	*EGFR*	7	5.5E+07	G	A	2.0%	40	1957	1997	p.G857R	missense_variant
3A	*EGFR*	7	5.5E+07	G	A	2.1%	41	1958	1999	p.A859T	missense_variant
3A	*MET*	7	1.2E+08	C	T	4.3%	24	535	559	p.Q826*	stop_gained
3A	*SMO*	7	1.3E+08	G	A	3.1%	62	1938	2000	p.C213Y	missense_variant
3A	*SMO*	7	1.3E+08	C	T	4.4%	87	1910	1997	p.A619V	missense_variant
3A	*ERBB4*	2	2.1E+08	C	T	4.6%	58	1192	1250	p.C593Y	missense_variant
3A	*BRAF*	7	1.4E+08	G	A	3.8%	44	1124	1168	p.Q461*	stop_gained
3A	*KRAS*	12	2.5E+07	C	T	4.2%	21	480	501	p.V114I	missense_variant
3A	*KRAS*	12	2.5E+07	G	A	4.1%	38	891	929	p.A18V	missense_variant
3A	*KRAS*	12	2.5E+07	C	T	4.1%	37	868	905	p.V7M	missense_variant
3B	*TP53*	17	7577132	C	T	2.0%	40	1959	1999	p.S269N	missense_variant

3B	*SMAD4*	18	4.9E+07	G	A	2.2%	44	1956	2000	p.R531Q	missense_variant
3B	*CDKN2A*	9	2.2E+07	G	A	3.0%	23	755	778	p.P114L	missense_variant
3B	*CDKN2A*	9	2.2E+07	G	A	3.7%	28	737	765	p.R107C	missense_variant
5A	*KRAS*	12	2.5E+07	C	A	2.0%	36	1796	1832	p.G12V	missense_variant
5B	*TP53*	17	7578417	C	CG	3.9%	76	1893	1969	p.E171fs	frameshift_variant,feature_elongation
6A	*TP53*	17	7579403	G	A	2.5%	49	1947	1996	p.S95F	missense_variant
6B	*APC*	5	1.1E+08	AG	GA	2.3%	42	1796	1840	p.K1462R	missense_variant
7A	*ABL1*	9	1.3E+08	G	A	3.7%	32	830	862	p.E334K	missense_variant
7A	*NOTCH1*	9	1.4E+08	C	T	4.3%	27	595	622	p.V2473I	missense_variant
7A	*NOTCH1*	9	1.4E+08	G	A	3.2%	21	627	648	p.Q2440*	stop_gained
7A	*RET*	10	4.4E+07	G	A	3.6%	19	503	522	p.V642I	missense_variant
7A	*RET*	10	4.4E+07	C	T	3.4%	27	760	787	p.A883V	missense_variant
7A	*PTEN*	10	9E+07	C	T	2.2%	44	1954	1998	p.R234W	missense_variant
7A	*TP53*	17	7573964	C	T	4.0%	71	1692	1763	p.A355T	missense_variant
7A	*TP53*	17	7577033	C	T	2.3%	27	1139	1166	p.G302E	missense_variant
7A	*TP53*	17	7577085	C	T	2.7%	32	1154	1186	p.E285K	missense_variant
7A	*TP53*	17	7577099	C	T	2.5%	30	1152	1182	p.R280K	missense_variant
7A	*TP53*	17	7577112	C	T	2.0%	23	1153	1176	p.A276T	missense_variant
7A	*TP53*	17	7577118	C	T	2.6%	30	1146	1176	p.V274I	missense_variant
7A	*TP53*	17	7577127	C	T	2.4%	28	1143	1171	p.E271K	missense_variant
7A	*TP53*	17	7577142	C	T	3.0%	35	1126	1161	p.G266R	missense_variant
7A	*TP53*	17	7578407	G	A	2.2%	18	785	803	p.R175C	missense_variant

7A	*TP53*	17	7578431	G	A	3.1%	25	775	800	p.Q167*	stop_gained
7A	*TP53*	17	7578475	G	A	3.0%	21	676	697	p.P152L	missense_variant
7A	*TP53*	17	7578476	G	A	3.6%	25	668	693	p.P152S	missense_variant
7A	*STK11*	19	1207021	C	T	2.9%	37	1243	1280	p.Q37*	stop_gained
7A	*STK11*	19	1223108	G	A	3.7%	26	670	696	p.E349K	missense_variant
7A	*EGFR*	7	5.5E+07	C	T	3.6%	48	1271	1319	p.P296L	missense_variant,splice_region_variant
7A	*EGFR*	7	5.5E+07	C	T	3.3%	25	744	769	p.S784F	missense_variant
7A	*CDKN2A*	9	2.2E+07	C	T	3.4%	15	427	442	p.G89S	missense_variant
7B	*BRAF*	7	1.4E+08	G	A	4.6%	82	1712	1794	p.Q456*	stop_gained
7B	*GNAQ*	9	8E+07	C	T	4.0%	79	1920	1999	p.C219Y	missense_variant
7B	*NOTCH1*	9	1.4E+08	G	A	2.4%	48	1952	2000	p.Q2440*	stop_gained
7B	*ATM*	11	1.1E+08	C	T	2.1%	42	1958	2000	p.R1730*	stop_gained
7B	*PTPN11*	12	1.1E+08	G	A	3.3%	66	1930	1996	p.M504I	missense_variant
7B	*RB1*	13	4.9E+07	G	A	4.4%	23	503	526	p.R320Q	missense_variant
7B	*IDH2*	15	9.1E+07	G	A	3.8%	65	1666	1731	p.R159C	missense_variant
7B	*TP53*	17	7577070	G	A	3.0%	60	1936	1996	p.R290C	missense_variant
7B	*TP53*	17	7577073	G	A	2.5%	50	1946	1996	p.L289F	missense_variant
7B	*TP53*	17	7577099	C	T	2.9%	59	1941	2000	p.R280K	missense_variant
7B	*TP53*	17	7577103	C	T	2.9%	57	1943	2000	p.G279R	missense_variant
7B	*VHL*	3	1E+07	G	A	2.3%	46	1951	1997	p.G144R	missense_variant
7B	*TP53*	17	7577604	C	T	2.4%	47	1952	1999	p.G226D	missense_variant
7B	*TP53*	17	7578400	G	A	3.0%	61	1939	2000	p.P177L	missense_variant

7B	*TP53*	17	7579377	G	A	2.2%	28	1221	1249	p.Q104*	stop_gained
7B	*SMAD4*	18	4.9E+07	G	A	3.5%	65	1792	1857	p.V333I	missense_variant
7B	*SMAD4*	18	4.9E+07	G	A	2.1%	39	1824	1863	p.R361H	missense_variant
7B	*SMARCB1*	22	2.4E+07	G	A	3.8%	65	1640	1705	p.V64I	missense_variant
7B	*SMARCB1*	22	2.4E+07	G	A	3.9%	42	1036	1078	p.R376K	missense_variant
7B	*PIK3CA*	3	1.8E+08	G	A	3.8%	49	1255	1304	p.A415T	missense_variant
7B	*FBXW7*	4	1.5E+08	C	T	3.4%	22	631	653	p.R465H	missense_variant
7B	*FBXW7*	4	1.5E+08	C	T	3.7%	24	622	646	p.G437R	missense_variant
7B	*APC*	5	1.1E+08	C	T	2.4%	47	1952	1999	p.Q1291*	stop_gained
7B	*EGFR*	7	5.5E+07	G	A	2.0%	40	1959	1999	p.G873E	missense_variant
7B	*BRAF*	7	1.4E+08	C	T	2.2%	17	752	769	p.V600M	missense_variant
7B	*CDKN2A*	9	2.2E+07	G	A	3.9%	25	616	641	p.T137I	missense_variant
7B	*CDKN2A*	9	2.2E+07	G	A	3.6%	29	769	798	p.R112C	missense_variant
7B	*CDKN2A*	9	2.2E+07	G	A	3.9%	28	696	724	p.R99W	missense_variant
8A	*TP53*	17	7578275	G	A	2.2%	43	1954	1997	p.Q192*	stop_gained
8A	*TP53*	17	7578428	G	A	2.0%	40	1960	2000	p.H168Y	missense_variant
8A	*TP53*	17	7579382	G	A	2.3%	37	1574	1611	p.T102I	missense_variant
9A	*BRAF*	7	1.4E+08	G	A	2.3%	18	754	772	p.T599I	missense_variant
9A	*KRAS*	12	2.5E+07	G	A	3.3%	27	794	821	p.A146V	missense_variant
9A	*KRAS*	12	2.5E+07	C	T	2.7%	33	1191	1224	p.V14I	missense_variant
9A	*KRAS*	12	2.5E+07	C	T	2.0%	24	1200	1224	p.G12S	missense_variant
9A	*CDKN2A*	9	2.2E+07	G	A	2.7%	33	1168	1201	p.A76V	missense_variant

9A	*CDKN2A*	9	2.2E+07	G	A	4.3%	51	1141	1192	p.P70S	missense_variant
9A	*RET*	10	4.4E+07	C	T	4.6%	33	682	715	p.P628L	missense_variant
9A	*RET*	10	4.4E+07	C	T	4.4%	23	503	526	p.A654V	missense_variant
9A	*RET*	10	4.4E+07	C	T	3.7%	54	1387	1441	p.S891L	missense_variant
9A	*RET*	10	4.4E+07	C	T	4.3%	44	974	1018	-	splice_region_variant,intron_variant
9A	*PTEN*	10	9E+07	G	A	3.3%	47	1393	1440	p.G132S	missense_variant
9A	*PTEN*	10	9E+07	G	A	3.8%	26	665	691	p.?_splice	splice_acceptor_variant
9A	*PTEN*	10	9E+07	G	A	2.0%	14	680	694	p.G165R	missense_variant,splice_region_variant
9A	*PTEN*	10	9E+07	C	T	3.5%	25	683	708	p.P169L	missense_variant
9A	*PTEN*	10	9E+07	C	T	2.0%	32	1591	1623	p.P246L	missense_variant
9A	*ATM*	11	1.1E+08	C	T	2.0%	39	1959	1998	p.R2691C	missense_variant
9A	*ATM*	11	1.1E+08	C	T	2.4%	30	1221	1251	p.R3047*	stop_gained
9A	*PTPN11*	12	1.1E+08	G	A	2.6%	50	1846	1896	p.E69K	missense_variant
9A	*FLT3*	13	2.9E+07	C	T	3.8%	35	882	917	p.D600N	missense_variant
9A	*RB1*	13	4.9E+07	T	C	4.0%	18	432	450	-	splice_region_variant,intron_variant
9A	*RB1*	13	4.9E+07	C	T	3.7%	16	420	436	p.L343F	missense_variant
9A	*RB1*	13	4.9E+07	C	T	3.1%	16	506	522	p.R358*	stop_gained
9A	*IDH2*	15	9.1E+07	C	T	3.8%	50	1267	1317	p.R159H	missense_variant
9A	*IDH2*	15	9.1E+07	G	A	2.1%	26	1225	1251	p.R140W	missense_variant
9A	*TP53*	17	7577033	C	T	2.9%	35	1178	1213	p.G302E	missense_variant
9A	*TP53*	17	7577061	C	T	3.6%	44	1185	1229	p.G293R	missense_variant
9A	*TP53*	17	7577120	C	T	2.4%	29	1204	1233	p.R273H	missense_variant

9A	*TP53*	17	7577142	C	T	2.7%	33	1189	1222	p.G266R	missense_variant
9A	*TP53*	17	7578205	C	T	2.6%	51	1946	1997	p.S215N	missense_variant
9A	*TP53*	17	7578263	G	A	2.3%	45	1948	1993	p.R196*	stop_gained
9A	*TP53*	17	7578371	C	T	3.4%	44	1268	1312	p.G187S	missense_variant,splice_region_variant
9A	*TP53*	17	7578419	C	T	2.2%	28	1242	1270	p.E171K	missense_variant
9A	*TP53*	17	7578427	T	G	2.4%	30	1241	1271	p.H168P	missense_variant
9A	*TP53*	17	7578457	C	T	2.1%	23	1082	1105	p.R158H	missense_variant
9A	*TP53*	17	7579373	C	T	2.6%	19	707	726	p.G105D	missense_variant
9A	*TP53*	17	7579395	G	A	2.7%	23	830	853	p.P98S	missense_variant
9A	*TP53*	17	7579403	G	A	2.1%	18	847	865	p.S95F	missense_variant
9A	*TP53*	17	7579422	G	A	3.8%	34	857	891	p.P89S	missense_variant
9A	*TP53*	17	7579436	G	A	3.0%	27	860	887	p.A84V	missense_variant
9A	*TP53*	17	7579464	G	A	3.8%	35	886	921	p.P75S	missense_variant
9A	*SMAD4*	18	4.9E+07	G	A	4.2%	59	1353	1412	p.V128M	missense_variant
9A	*SMAD4*	18	4.9E+07	G	A	3.9%	35	858	893	p.D424N	missense_variant
9A	*PIK3CA*	3	1.8E+08	G	A	4.4%	31	668	699	p.?_splice	splice_donor_variant
9A	*PIK3CA*	3	1.8E+08	G	A	3.8%	48	1227	1275	p.E321K	missense_variant
9A	*PIK3CA*	3	1.8E+08	C	T	2.0%	33	1656	1689	p.A1020V	missense_variant
9A	*FGFR3*	4	1808898	C	T	4.1%	54	1255	1309	p.T777I	missense_variant
9A	*FGFR3*	4	1808981	C	T	4.5%	45	959	1004	p.R805W	missense_variant
9A	*KIT*	4	5.6E+07	G	A	3.1%	41	1289	1330	p.E562K	missense_variant
9A	*KIT*	4	5.6E+07	C	T	2.1%	28	1296	1324	p.L576F	missense_variant

9A	*KIT*	4	5.6E+07	C	T	2.5%	34	1306	1340	p.P577S	missense_variant
9A	*KIT*	4	5.6E+07	C	T	2.3%	45	1952	1997	p.T670I	missense_variant
9A	*KDR*	4	5.6E+07	C	T	3.8%	55	1403	1458	p.G1348E	missense_variant
9A	*KDR*	4	5.6E+07	G	A	4.4%	64	1389	1453	p.P1345L	missense_variant
9A	*KDR*	4	5.6E+07	C	T	3.9%	77	1921	1998	p.V1294M	missense_variant
9A	*KDR*	4	5.6E+07	G	A	4.7%	69	1394	1463	p.S884F	missense_variant
9A	*FBXW7*	4	1.5E+08	C	T	3.5%	34	928	962	p.E449K	missense_variant
9A	*FBXW7*	4	1.5E+08	C	T	3.6%	35	927	962	p.V445M	missense_variant
9A	*APC*	5	1.1E+08	C	T	3.5%	37	1012	1049	p.P1319L	missense_variant
9A	*APC*	5	1.1E+08	C	T	2.5%	34	1306	1340	p.A1366V	missense_variant
9A	*EGFR*	7	5.5E+07	C	T	3.6%	31	840	871	p.P281S	missense_variant
9A	*EGFR*	7	5.5E+07	C	T	3.8%	44	1118	1162	p.A289V	missense_variant
9A	*EGFR*	7	5.5E+07	G	A	2.4%	47	1953	2000	p.G719S	missense_variant
9A	*EGFR*	7	5.5E+07	G	A	4.2%	43	993	1036	p.G729E	missense_variant,splice_region_variant
9A	*EGFR*	7	5.5E+07	G	A	4.6%	48	992	1040	p.E736K	missense_variant
9A	*EGFR*	7	5.5E+07	C	T	4.1%	43	1006	1049	p.A750V	missense_variant
9A	*EGFR*	7	5.5E+07	G	A	2.4%	37	1531	1568	p.G810D	missense_variant
9A	*SMO*	7	1.3E+08	C	T	3.5%	69	1930	1999	p.P610S	missense_variant
9A	*IDH1*	2	2.1E+08	C	T	2.1%	42	1958	2000	p.R132H	missense_variant
9A	*FGFR1*	8	3.8E+07	G	A	4.3%	34	764	798	p.S158L	missense_variant
9A	*CDKN2A*	9	2.2E+07	C	T	3.9%	23	567	590	p.R131H	missense_variant
9A	*CDKN2A*	9	2.2E+07	G	A	3.6%	21	568	589	p.R131C	missense_variant

10A	*APC*	5	1.1E+08	AG	GA	2.2%	41	1817	1861	p.K1462R	missense_variant
11A	*TP53*	17	7577018	C	T	2.0%	29	1399	1428	p.?_splice	splice_donor_variant
11A	*APC*	5	1.1E+08	AG	GA	2.0%	37	1811	1850	p.K1462R	missense_variant
13B	*TP53*	17	7578380	C	T	2.0%	40	1959	1999	p.D184N	missense_variant
14A	*TP53*	17	7577539	GG	AA	2.6%	21	775	796	p.R248W	missense_variant
14A	*TP53*	17	7579382	G	A	2.2%	42	1888	1930	p.T102I	missense_variant
14B	*APC*	5	1.1E+08	C	T	2.5%	49	1951	2000	p.Q1367*	stop_gained
15A	*RET*	10	4.4E+07	G	A	4.2%	36	827	863	-	splice_region_variant,intron_variant
15A	*IDH2*	15	9.1E+07	C	T	2.2%	30	1350	1380	p.R140Q	missense_variant
15A	*TP53*	17	7577132	C	T	2.2%	33	1441	1474	p.S269N	missense_variant
15A	*TP53*	17	7578370	C	T	3.6%	40	1084	1124	p.?_splice	splice_donor_variant
15A	*TP53*	17	7578413	C	G	2.2%	25	239	1125	p.V173L	missense_variant
15A	*TP53*	17	7578431	G	A	2.5%	29	1109	1138	p.Q167*	stop_gained
15A	*TP53*	17	7578476	G	A	2.6%	25	947	972	p.P152S	missense_variant
15A	*TP53*	17	7579377	G	A	3.1%	19	594	613	p.Q104*	stop_gained
15A	*GNA11*	19	3118925	G	A	3.5%	27	745	772	p.M203I	missense_variant
15A	*EGFR*	7	5.5E+07	C	T	2.3%	27	1167	1194	p.S784F	missense_variant
15A	*CDKN2A*	9	2.2E+07	C	T	2.5%	10	396	406	p.R131H	missense_variant
15A	*CDKN2A*	9	2.2E+07	G	A	2.5%	27	1063	1090	p.A76V	missense_variant
17A	*APC*	5	1.1E+08	G	T	4.8%	96	1898	1994	p.E1286*	stop_gained
17B	*APC*	5	1.1E+08	AG	GA	2.1%	39	1858	1901	p.K1462R	missense_variant
19A	*KRAS*	12	2.5E+07	G	A	2.1%	22	1002	1024	p.A146V	missense_variant

19A	*ABL1*	9	1.3E+08	G	A	4.3%	22	488	510	p.G321E	missense_variant
19A	*NOTCH1*	9	1.4E+08	G	A	3.6%	20	537	557	p.S1589F	missense_variant
19A	*IDH2*	15	9.1E+07	C	T	2.0%	29	1387	1416	p.R140Q	missense_variant
19A	*STK11*	19	1220499	G	A	4.3%	20	444	464	p.A198T	missense_variant
19A	*EGFR*	7	5.5E+07	G	A	2.2%	17	754	771	p.V774M	missense_variant
19A	*EGFR*	7	5.5E+07	G	A	3.2%	63	1937	2000	p.G873E	missense_variant
1B	*APC*	5	1.1E+08	AG	GA	2.3%	42	1791	1835	p.K1462R	missense_variant
20A	*SMARCB1*	22	2.4E+07	C	A	4.5%	59	1242	1301	p.T72K	missense_variant
20A	*CDKN2A*	9	2.2E+07	G	A	2.0%	13	639	652	p.A102V	missense_variant
21A	*CDKN2A*	9	2.2E+07	C	T	2.2%	12	541	553	p.?_splice	splice_acceptor_variant
22A	*TP53*	17	7577099	C	T	2.1%	41	1959	2000	p.R280K	missense_variant
22A	*TP53*	17	7578263	G	A	2.2%	44	1953	1997	p.R196*	stop_gained
22A	*TP53*	17	7579394	G	A	2.3%	32	1383	1415	p.P98L	missense_variant
22A	*PIK3CA*	3	1.8E+08	G	A	3.2%	63	1931	1994	p.A1066T	missense_variant
23B	*TP53*	17	7577138	C	T	2.0%	39	1952	1991	p.R267Q	missense_variant
23B	*CDKN2A*	9	2.2E+07	C	T	2.1%	25	1192	1217	p.R131H	missense_variant
24B	*APC*	5	1.1E+08	AG	GA	2.0%	38	1839	1878	p.K1462R	missense_variant
25A	*FGFR3*	4	1806083	G	A	3.5%	14	391	405	p.E368K	missense_variant
25A	*FGFR3*	4	1806170	C	T	3.6%	12	321	333	p.R397C	missense_variant
26A	*TP53*	17	7577557	AG	A	2.5%	30	1167	1197	p.C242fs	frameshift_variant,missense_variant,feature_truncation
27A	*APC*	5	1.1E+08	AG	GA	2.2%	42	1826	1871	p.K1462R	missense_variant
27A	*JAK2*	9	5073770	G	T	2.5%	50	1946	1996	p.V617F	missense_variant

29A	*KRAS*	12	2.5E+07	C	T	2.3%	44	1849	1893	p.G12D	missense_variant
29B	*APC*	5	1.1E+08	C	T	4.7%	94	1906	2000	p.R876*	stop_gained

A :specimens were collected by biopsy before nCRT
B : specimens were collected by surgery after nCRT
*indicated stop codon.

Supplementary Table S5. Number of genetic variants detected in patients with rectal carcinoma

			Synonymous			Nonsynonymous	
Tumor regression Grade	Patients	polymorphism	mutation	total	polymorphism	mutation	total
1	1A	6	0	6	3	1	4
1	1B	6	0	6	3	0	3
1	2A	4	5	9	4	4	8
1	2B	4	2	6	3	0	3
0	3A	9	15	24	5	28	33
0	3B	8	1	9	3	0	3
0	4A	5	0	5	3	3	6
0	4B	5	0	5	3	0	3
2	5A	7	0	7	2	4	6
2	5B	7	0	7	2	0	2
0	6A	6	0	6	3	3	6
0	6B	6	0	6	3	0	3
1	7A	6	1	7	4	4	8
1	7B	6	0	6	4	1	5
0	8A	6	0	6	2	3	5
0	8B	6	0	6	2	0	2

1	9A	7	1	8	4	5	9
1	9B	4	0	4	4	0	4
2	10A	5	0	5	4	2	6
2	10B	6	0	6	4	0	4
1	11A	5	0	5	3	2	5
1	11B	5	0	5	3	0	3
2	13A	6	1	7	1	3	4
2	13B	5	1	6	1	0	1
3	14A	6	0	6	3	2	5
3	14B	6	0	6	3	0	3
2	15A	6	1	7	3	3	6
2	15B	7	1	8	3	0	3
0	17A	6	0	6	4	1	5
0	17B	6	0	6	4	0	4
3	18A	6	0	6	4	3	7
3	18B	6	0	6	4	4	8
0	19A	8	0	8	3	4	7
0	19B	8	0	8	3	0	3
2	20A	3	0	3	3	1	4

2	20B	3	0	3	3	1	4
0	21A	5	0	5	5	2	7
0	21B	N.A	N.A	N.A	N.A	N.A	N.A
2	22A	4	0	4	3	1	4
2	22B	4	0	4	3	0	3
1	23A	5	0	5	3	3	6
1	23B	5	0	5	3	2	5
1	24A	5	0	5	3	6	9
1	24B	5	0	5	3	1	4
1	25A	7	0	7	2	2	4
1	25B	7	0	7	2	2	4
0	26A	7	1	8	2	2	4
0	26B	N.A	N.A	N.A	N.A	N.A	N.A
1	27A	5	0	5	2	4	6
1	27B	5	0	5	2	1	3
0	28A	6	0	6	3	2	5
0	28B	N.A	N.A	N.A	N.A	N.A	N.A
2	29A	9	0	9	3	4	7
2	29B	9	0	9	3	3	6

1	30A	5	0	5	3	4	7
1	30B	5	0	5	3	1	4
1	31A	6	0	6	3	8	11
1	31B	6	0	6	3	0	3

A : specimens were collected by biopsy before nCRT
B : specimens were collected by surgery after nCRT
N.A: Not Applicable

Supplementary Table S6. The associations between gene mutation status responses from patients with rectal carcinoma.

	Tumor regression				
	Grade 0 (n=9)		Grade 1-3 (n=20)		
	n	(%)	n	(%)	p value
**ABL 1**					n/a
WT	9	(31.0)	20	(69.0)	
Mutation	0	(0.0)	0	(0.0)	
**AKT 1**					n/a
WT	9	(31.0)	20	(69.0)	
Mutation	0	(0.0)	0	(0.0)	
**ALK**					0.532[b]
WT	8	(29.6)	19	(70.4)	
Mutation	1	(50.0)	1	(50.0)	
**APC**					0.431[b]
WT	2	(20.0)	8	(80.0)	
Mutation	7	(36.8)	12	(63.2)	
**ATM**					1.000[b]
WT	8	(33.3)	16	(66.7)	
Mutation	1	(20.0)	4	(80.0)	
**BRAF**					0.633[b]
WT	8	(34.8)	15	(65.2)	

Mutation	1	(16.7)	5	(83.3)	
**CDH1**					n/a
WT	9	(31.0)	20	(69.0)	
Mutation	0	(0.0)	0	(0.0)	
**CDKN2A**					0.675[b]
WT	7	(35.0)	13	(65.0)	
Mutation	2	(22.2)	7	(77.8)	
**CHL**					n/a
WT	9	(31.0)	20	(69.0)	
Mutation	0	(0.0)	0	(0.0)	
**CTNNB1**					1.000[b]
WT	8	(30.8)	18	(69.2)	
Mutation	1	(33.3)	2	(66.7)	
**EGFR**					1.000[b]
WT	7	(31.8)	15	(68.2)	
Mutation	2	(28.6)	5	(71.4)	
**ERBB2**					n/a
WT	9	(31.0)	20	(69.0)	
Mutation	0	(0.0)	0	(0.0)	
**ERBB4**					1.000[b]
WT	8	(30.8)	18	(69.2)	
Mutation	1	(33.3)	2	(66.7)	
**FBXW7**					0.339[b]

WT	6	(26.1)	17	(73.9)	
Mutation	3	(50.0)	3	(50.0)	
**FGFR1**					0.633[b]
WT	7	(29.2)	17	(70.8)	
Mutation	2	(40.0)	3	(60.0)	
**FGFR3**					1.000[b]
WT	8	(30.8)	18	(69.2)	
Mutation	1	(33.3)	2	(66.7)	
**FLT3**					1.000[b]
WT	8	(32.0)	17	(68.0)	
Mutation	1	(25.0)	3	(75.0)	
**GNA11**					1.000[b]
WT	9	(33.3)	18	(66.7)	
Mutation	0	(0.0)	2	(100.0)	
**GNAQ**					0.310[b]
WT	8	(28.6)	20	(71.4)	
Mutation	1	(100.0)	0	(0.0)	
**IDH2**					1.000[b]
WT	9	(32.1)	19	(67.9)	
Mutation	0	(0.0)	1	(100.0)	
**JAK2**					n/a
WT	9	(31.0)	20	(69.0)	
Mutation	0	(0.0)	0	(0.0)	

**KDR**					1.000[b]
WT	8	(32.0)	17	(68.0)	
Mutation	1	(25.0)	3	(75.0)	
**KIT**					1.000[b]
WT	8	(33.3)	16	(66.7)	
Mutation	1	(20.0)	4	(80.0)	
**KRAS**					0.245[b]
WT	3	(20.0)	12	(80.0)	
Mutation	6	(42.9)	8	(57.1)	
**MET**					**<0.001[b]**
WT	2	(9.5)	19	(90.5)	
Mutation	7	(87.5)	1	(12.5)	
**NOTCH1**					n/a
WT	9	(31.0)	20	(69.0)	
Mutation	0	(0.0)	0	(0.0)	
**PDGFRA**					n/a
WT	9	(31.0)	20	(69.0)	
Mutation	0	(0.0)	0	(0.0)	
**PIK3CA**					0.107[b]
WT	8	(42.1)	11	(57.9)	
Mutation	1	(10.0)	9	(90.0)	
**PTEN**					0.633[b]
WT	8	(34.8)	15	(65.2)	

Mutation	1	(16.7)	5	(83.3)	
**PTPN11**					0.280[b]
WT	9	(36.0)	16	(64.0)	
Mutation	0	(0.0)	4	(100.0)	
**RB1**					1.000[b]
WT	8	(30.8)	18	(69.2)	
Mutation	1	(33.3)	2	(66.7)	
**RET**					1.000[b]
WT	8	(33.3)	16	(66.7)	
Mutation	1	(20.0)	4	(80.0)	
**SMAD4**					0.633[b]
WT	8	(34.8)	15	(65.2)	
Mutation	1	(16.7)	5	(83.3)	
**SMARCB1**					0.532[b]
WT	8	(29.6)	19	(70.4)	
Mutation	1	(50.0)	1	(50.0)	
**SMO**					n/a
WT	9	(31.0)	20	(69.0)	
Mutation	0	(0.0)	0	(0.0)	
**STK11**					1.000[b]
WT	8	(30.8)	18	(69.2)	
Mutation	1	(33.3)	2	(66.7)	
**TP53**					0.532[b]

WT	1	(50.0)	1	(50.0)
Mutation	8	(29.6)	19	(70.4)

[a]*p-value* is estimated by chi-square test
[b]*p-value* is estimated by ***Fisher's test***
